# Microwave Sensing and Imaging Technology in Food Applications: A Comprehensive Review

**DOI:** 10.1111/1541-4337.70220

**Published:** 2025-07-08

**Authors:** Aysenur Betul Bilgin, Pervin Basaran

**Affiliations:** ^1^ Department of Food Engineering, Faculty of Chemical and Metallurgical Engineering Istanbul Technical University Maslak Istanbul Türkiye

**Keywords:** detection, food quality, image, microwave, non‐destructive, sensor

## Abstract

Microwave sensing (MWS) and imaging (MWI) technologies have gained significant attention as non‐destructive methods for assessing food quality, ensuring safety, and verifying authenticity. This review provides a comprehensive evaluation of MW‐based systems in food applications, integrating both theoretical foundations and practical implementations. The fundamental principles of MW technology, including its theoretical background, sensing mechanisms, and imaging techniques, are first discussed. The review then explores the applications of MW sensing and imaging in food analysis, encompassing contamination detection (e.g., foreign bodies, microorganisms, and toxins), moisture content evaluation, adulteration detection, quality control, and compositional assessment. Furthermore, the advantages and limitations of MW systems for food applications are critically analyzed, along with an overview of commercial MW‐based technologies, relevant patent developments, and ongoing international research initiatives. Finally, the future potential of MWS and MWI in the food industry is discussed, emphasizing their role in advancing real‐time, non‐invasive quality monitoring and strengthening food integrity. This review aims to provide valuable insights into the current state and future directions of MW‐based food inspection technologies.

## 1 Introduction

Microwave (MW) is a region in the electromagnetic (EM) spectrum with a frequency range of 300–300 GHz (*λ* = 1 mm–1 m) between the infrared and radiofrequency regions on the spectrum (Pozar 2021). It consists of various bands, including ultra‐high frequency (UHF) (300 MHz–3 GHz), L (1–2 GHz), S (2–4 GHz), C (4–8 GHz), X (8–12 GHz), Ku (12–18 GHz), K (18–27 GHz), Ka (27–40 GHz), V (40–75 GHz), and W (75–110 GHz), and millimeter (mm) wave (3–300 GHz) band (Pozar 2021; Jeong et al. 2024). The MW region can also be categorized as traditional MW (decimeter and centimeter waves) and mm‐wave bands. The classification of bands is determined by factors, including resolution, penetration power, and other factors that influence sample interaction.

MW is widely used in various industries, including composites (Li, Wang, et al. 2021; Saif ur Rahman et al. 2024), medical treatments (Chandra et al. 2015; Gartshore et al. 2021; Halim et al. 2022), and food processing (Sisquella et al. 2013; Eissa et al. 2021; Costa et al. 2021). In food processing, MW can be applied for heating purposes (i.e., thermal treatment), sterilization, and extraction, as well as non‐destructive testing (NDT).

MW testing has been used since the early 1950s and can be classified as MW sensing or spectroscopy (MWS) and imaging (MWI). In recent years, interest in this method has increased. The German Society of Non‐Destructive Testing established an expert committee for MW and terahertz (THz) testing procedures in 2011, followed by the American Society for Non‐Destructive Testing in 2014 and the British Institute of Non‐Destructive Testing in 2015 (Li, Wang, et al. 2021). Boeing Company established a new MW laboratory in 2017 for NDT of potential flaws in coatings, surfaces, and structures, recognizing MW testing as its own NDT method (Careaga 2017). MW systems have also grown significantly in conducting R&D activities in food applications worldwide. They have recently gained widespread acceptance within the food industry for food safety monitoring, including adulteration detection (Soltani Firouz et al. 2021, Soltani Firouz et al. 2022), contamination (Tobon Vasquez et al. 2019), fruit and vegetable maturity (Garvin et al. 2023), and foreign body detection (Urbinati et al. 2020; Li et al. 2022; Zeni et al. 2023). The increasing interest in these technologies for food safety evaluation is largely due to their advantages such as non‐invasive, minimal or no sample preparation, fast measurement, low cost, and the ability to perform at‐line, online, and in‐line analysis (Meng et al. 2017; Tobon Vasquez et al. 2020; Liu et al. 2022; Jeong et al. 2024). We have reviewed and identified international projects relative to MW detection systems in food applications (Table 1). These international research projects, including BEST‐Food (Italy), EPSRC‐funded studies (UK), and multiple initiatives in China, focus on developing MWI and MWS for non‐destructive food quality, safety, and authenticity assessment. With advancements in artificial intelligence (AI) and real‐time monitoring, MW‐based food inspection technologies are expected to grow in the next 5–10 years, enhancing precision, automation, and industrial adoption. Table 2 demonstrates organizations (companies, universities, and research institutes) that have filed MWS and MWI patents for food applications. The table presents various MW systems and methods (dielectric spectroscopy, tomographic imaging, etc.), including insecticide detection and non‐destructive food quality measurements, with patents from companies like Huawei, Mitsubishi, and General Electric, spanning from 2007 to 2024. Moreover, our web‐based research revealed commercial MW systems producing companies for food‐related industries (Table 3). To the best of our knowledge, the companies, including Keysight Technologies, Food Radar, MicroRadar, and Vertigo Technologies, have been continuing to develop MW‐based systems for food quality assessment, safety assurance, and the detection of contamination and moisture content (MC). Although these advancements are expected to accelerate industrial adoption, relatively high cost, large size, and power requirements of MW systems may continue to pose significant barriers to large‐scale implementation in high‐throughput food processing plants. This still presents a gap between technological innovation and its practical deployment in the food industry.

**TABLE 1:** Projects related to microwave systems for food applications.

Project title	MW systems	Year	Supported by	Grant no.	References
BEST‐Food, Broadband Electromagnetic Sensing Technologies for Food Quality and Security Assessment	MWI	2017	Italian Ministry of University and Research	20179FLH4A	Urbinati et al. (2020), Ricci et al. (2021a), Ricci et al. (2022), Bellizzi et al. (2022), Zeni et al. (2023)
N.A.	MWS	N.A.	Natural Science Foundation of Jiangsu Province	BK20200427	Li et al. (2022)
			Shuangchuang Project of Jiangsu Province	KFR20020	
			Fundamental Research Funds for the Central Universities	NS2020019	
			National Natural Science Foundation of China	52105552	
N.A.	MWS—OECP	N.A.	National Natural Science Foundation of China	31671935	Zhu et al. (2018)
N.A.	MWS—free space method	N.A.	National Natural Science Foundation of China	61874050	Xu et al. (2022), Bai et al. (2024)
			Postgraduate Research & Practice Innovation Program of Jiangsu Province	SJCX21_1690	
N.A.	MWS—free space method	N.A.	National Key Research and Development Program of China	2017YFC1600603	Deng et al. (2023)
N.A.	MWI	N.A.	UK's Engineering and Physical Sciences Research Council	EP/R013918/1	Ghavami et al. (2021)
			Innovate UK	103920	

Abbreviations: MWI, microwave imaging; MWS, microwave sensing; N.A., not assessed; OECP, open‐ended coaxial probe.

**TABLE 2:** Microwave systems related to food applications at the US Patent Office.

Innovation (method, system, apparatuses, devices, product, application, etc.)	System features	Companies	No. of patents	Patent date
MW systems				
MW antenna probe	Providing a solution for a non‐destructive method for probing an MW antenna patch on a printed circuit board and direct access to the transmitting waveform	Huawei Technologies Co. Ltd. (Shenzhen/CN)	1	2024
Dielectric spectroscopy sensing apparatus and method of use	Consists of a test volume, electrodes for receiving and delivering radio frequency signals, and a floating electrode for detecting fluid inlet	Xatek Inc. (Chagrin Falls, OH/US)	2	2024
System and method for tomographic imaging	Utilizing wave‐field measurements to reconstruct an object's internal structure, recursively adding frequencies to minimize differences between measurements and synthesized wave‐fields generated by a neural network operator	Mitsubishi Electric Research Laboratories Inc. (Cambridge, MA/US)	1	2024
Portable dielectric spectroscopy device	A portable DS device includes a device housing, device‐side electrical contacts, a computing system, and a removable sensor receiver assembly. It communicates with a fluid sensing apparatus and device‐side contacts	Xatek Inc. (Chagrin Falls, OH/US)	1	2024
Non‐invasive sensor and method of use	A method for measuring temperature and composition of an aqueous target involves a transmitter and receiver mechanism, antennas, and a signal processor. The method involves irradiating the target with electromagnetic radiation signals, receiving attenuated signals, and calculating the attenuation of the transmitted signal	Agresearch Limited (Hamilton/NZ)	1	2007
Detection for food applications				
Detecting and imaging using dielectric tomography	MW signals are used for sensing, detecting, characterizing, and imaging dielectric objects. A dielectric tomography system (10 MHz–300 GHz) involves positioning an object in an EM field, determining permittivity information, and computing images based on permittivity and calibration information. This method allows for accurate detection and characterization of dielectric objects	Omnizare Imaging Inc. (Waltham, MA/US)	2	2024
Measurement and imaging instruments and beamforming method	A high‐speed, accurate measurement and imaging instrument with a reception unit and main body for beamforming processing. It generates a reception signal from a wave and performs lateral modulation, Hilbert transform, and partial derivative processing to generate analytic signals. The instrument also performs lateral modulation and multi‐dimensional processing for accurate calculations	Chikayoshi Sumi (Saitama/JP)	1	2024
Insecticide detection device	Uses microwave circuitry, antenna, power detector, and dosage level indicator to generate and transmit a reflected microwave signal, indicating the insecticide's level on the surface	Liverpool John Moores University (Liverpool/GB)	1	2024
Food preparation apparatus and method	Consists of a compartment, a dielectric sensor, a data storage device, and a processor arrangement. The processor determines the dielectric property of a food product, retrieves seasoning data, and generates seasoning instructions for adding condiments to the product. The method also includes a method of automatically generating seasoning instructions for adding multiple condiments during food preparation	Koninklijke Philips N.V. (Eindhoven/Nl)	1	2020
System and method for detection of nutritional parameters in food items	Measures nutritional parameters of food items include a holding cavity, sensor assembly, transmitter at least one receiver antenna, switches, and a processing unit. The transmitter antenna is configured to transmit signals to a food item in the holding cavity. The system transmits signals to the food item, receives responses, and connects to a power source. The system also includes a processing unit for determining nutritional parameters	General Electric Company (Schenectady, NY/US)	1	2016
Systems and methods for non‐destructively measuring calorie contents of food items	Measures the calorie content of a food item using ultra‐wide band (UWB) signals. The device includes a holder substrate, transmitter antenna, first and second receiver (planar) antennas, and a transmitter antenna for reflected and propagated UWB signals, allowing for accurate measurement of fat and water contents	General Electric Company (Schenectady, NY/US)	1	2016
Microwave sensor and algorithm for moisture and density determination	Determination of instantaneous bulk density and moisture content in agricultural commodities, using an inexpensive circuit for real and imaginary complex permittivity	The United States of America, as represented by the Secretary of Agriculture, (Washington, DC/US)	1	2014
System and method for detecting foreign objects in a product	The invention relates to a system for detecting changes in product material composition. It involves a microwave and ultrasound transmitters emitting signals, creating a density displacement within the product. The system measures the attenuation and runtime between the signals, comparing it with a previously determined attenuation or runtime to determine a possible material composition change	Food Radar System in Sweden AB (Goteborg/SE)	1	2009
MW method and system for material inspection	Inspects mediums, particularly food products transferred by a conveyor belt, in real time. MW includes a transmitter, receiver, scanner, waveform extractor, and CPU for transmitting, receiving, directing, extracting, and analyzing data A method for inspecting a food product involves transmitting a microwave through the product, receiving it in multiple channels of a receiving waveguide array, and analyzing the received microwave to inspect the food product using a signal analyzer	1 M International Corporation (Waterloo/CA)	1	2007

**TABLE 3:** Commercial companies developing and marketing microwave‐based systems for food applications.

Company	Claim	Websites
Agilent, Keysight Technologies, US	Provides VNA, PNA, portable/handheld (e.g., N9951A FieldFox MW analyzer), and benchtop MW and radiofrequency analyzers and their software	https://www.keysight.com/zz/en/home.html
MicroRadar, Union of the companies from different countries	MW moisture meter for measuring moisture of fluid, solid, and bulk materials. It can measure moisture (0%–100%), with accuracy from 0.02% up to 2%, in laboratories and in industrial processes	https://www.microradar.com/
Food Radar, SE	Provides to detect of foreign body (e.g., glass, metal, stones, wood splinters, hard and soft plastics, rubber, extraneous vegetable matter, fruit stones, and starch lumps) Emulsions, and pumpable products and measures the dielectric properties of the food Useful for food applications such as baby food, tomato processing, fruit preparation, desserts, soups, sauces, dressings, wet condiments, and relishes	https://www.foodradar.com/
NanoVNA, CN	Handheld vector network analyzer—nanoVNASaver software (50 kHz–1500 MHz) Detect food contaminants such as plastic, fragment fruit stones, glass, and wood during the manufacture of pumpable food products	https://nanorfe.com/nanovna‐v2.html
Maury Microwave, US	Prodives adapters, cable assemblies, and attenuators; coaxial and waveguide VNA calibration kits, calibration standards, and calibration systems; turnkey characterization solutions, including measurement and modeling software	https://maurymw.com/
Vertigo Technologies, NL	Provides hardware and software tools for MW and mm wave test (VNA calibration, microwave material testing, mm‐wave large signal characterization, and extreme impedance measurements) “Fresco MWS” provides detecting internal quality (ripeness stage, internal defects, and shelf life, Brix, dry matter, firmness, titratable acidity, oil content, internal defects) of fresh fruits (tested on mangoes, avocados, pears, apples, tomatoes) with non‐destructive MWS system	https://vertigo‐tech.com/ https://fresco.vertigo‐tech.com/
Sartorius LMA200, DE	MW based moisture analyzer for super‐fast moisture analysis in liquid and pasty samples with a moisture content ranging from approx. 8% to 100%	https://www.sartorius.com/en
TEWS, DE	Inline moisture measuring method	https://www.tewsworks.com/en
MAC Instruments, US	The MAC116 moisture analyzer, designed for continuous conveyor ovens for meat and poultry cooking, is successfully operating in numerous global cooking installations	https://macinstruments.com/

Abbreviation: MWS, microwave sensing.

## 2 Microwave Systems: Principles and Methods

### 2.1 Theoretical Background

MW systems are crucial for analyzing the dielectric properties of food samples, such as permittivity (Trabelsi and Nelson 2016). These properties are critical in understanding how food interacts with MW energy. The permittivity (*ε*) of a food sample is described by the real (*ε*′) and imaginary (*ε*″) components (Li et al. 2021). The real part represents the food's ability to store MW energy, whereas the imaginary part accounts for energy loss during absorption. The loss tangent (tan *δ*), which is the ratio of *ε*″ to ε′, is used to characterize the sample's energy (Mehdizadeh 2010), as shown in the following equation:

(1)

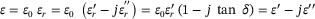




**FIGURE 1:**
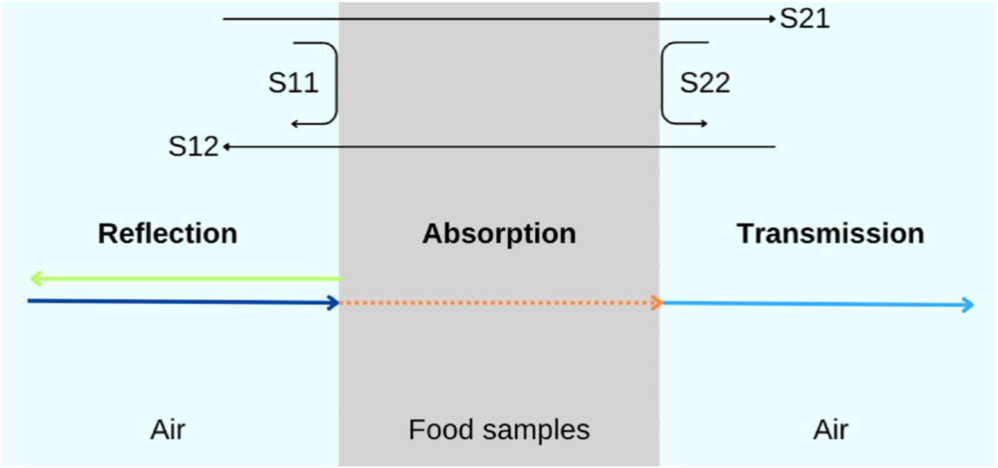
Interaction between MW energy and food samples.

The dielectric properties help determine the food's response to MW energy, such as energy absorption, reflection, and transmission (Figure 1), based on its components like water, carbohydrates, proteins, and lipids (Mehdizadeh 2010; Chandra et al. 2015). Scattering parameters (*S*‐parameters), such as input reflection coefficient/return losses (*S*_11_), output reflection (*S*_22_), forward transmission/insertion losses (*S*_21_), and reverse transmission (*S*_12_) (Figure 1), are commonly used to measure electrical signal propagation and reflection/transmission losses in food samples (Vp and Susan 2023).

**FIGURE 2:**
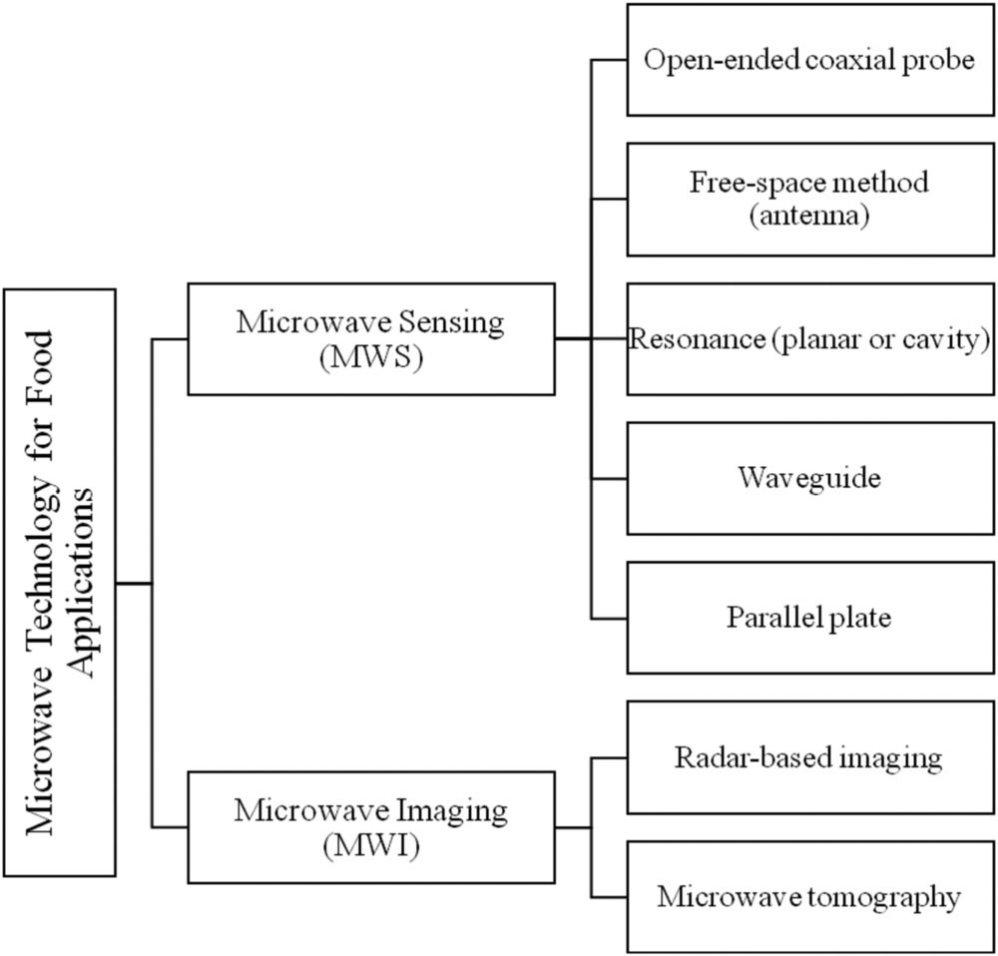
Microwave technology for food applications.

The MW systems (MWS and MWI) consist of hardware and software parts. These systems’ hardware includes antennas (such as horn, Vivaldi, patch, and microstrip) or sensors (such as coaxial probes and waveguides) and network analyzers. These components work synergistically with sophisticated software to analyze reflected and transmitted signals by measuring *S*‐parameters (*S*_11_, *S*_22_, *S*_21_, and *S*_12_), dielectric constant, and impedance. This detailed analysis can produce 1D, high‐resolution 2D, or 3D images that map the dielectric properties of the food samples, thereby linking the food's composition—including water, carbohydrates, proteins, and lipids—to its EM response with the software part (Chandra et al. 2015; Javanbakht et al. 2021a). Conclusively, MWS and MWI systems rely on detailed dielectric measurements and advanced imaging techniques (Figure 2). Thus, these systems can assess food composition and detect contaminants in real time, offering significant applications in food safety and quality assurance.

### 2.2 Microwave Sensing

MWS, or dielectric or microwave spectroscopy, is used for food safety and quality control by comparing a sample's dielectric constant, *S*‐parameters, or impedance (Guo et al. 2010; Chee et al. 2014; Nakonieczna et al. 2016; Li et al. 2021; Zidane et al. 2021). This technique can be categorized into near‐ and far‐field approaches based on the distance between sensors and the samples for food application. Near field refers to the field of an open‐ended coaxial probe (OECP), resonator (planar or cavity), and parallel plate (PP) methods. Far‐field refers to the field of operation for most antennas (Li et al. 2021). Each technique in near‐ and far‐field methods operates across different frequency ranges. This Section 2.2 provides an overview of various techniques, focusing on the OECP technique and free‐space method, which are the most commonly used methods for food applications.

#### 2.2.1 Open‐Ended Coaxial Probe

**FIGURE 3:**
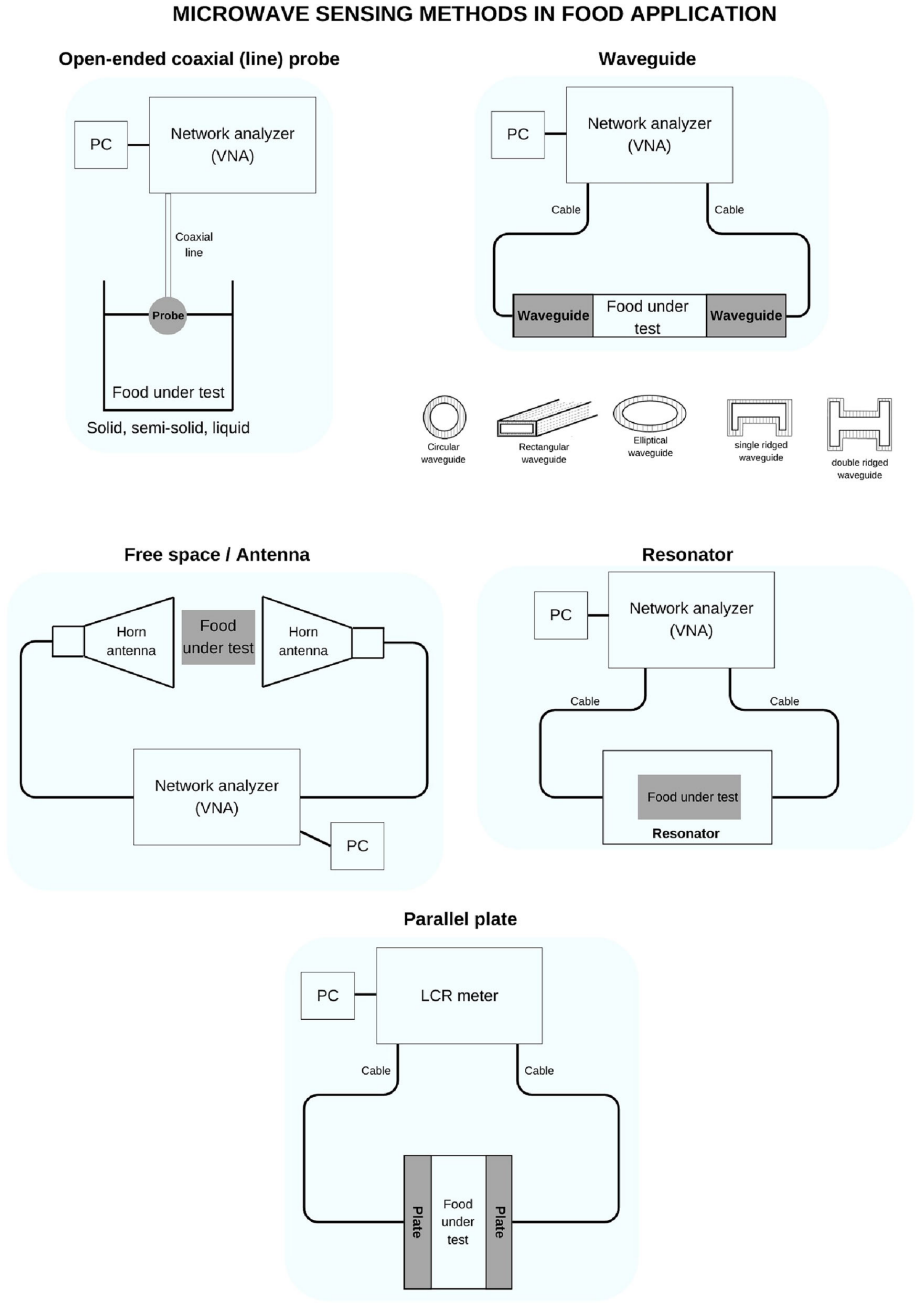
Microwave sensing (MWS) system in food application.

The OECP method is a truncated transmission line technique and offers a wide frequency range and simplicity, as a technique of MWS. The system setup consists of a VNA enabling measurements between 10 MHz and 50 GHz (UHF—Ka bands), a coaxial probe, food under test (FUT), and software, as shown in Figure 3. The reflected signals at different frequencies are measured and then converted into complex permittivity values by software (Gioia et al. 2018). It does not require sample preparation to assess food samples’ dielectric properties (i.e., permittivity). It involves immersing the probe into a liquid or touching it to a solid or semi‐solid face. Thus, this method is best suited for measuring the dielectric properties of homogeneous liquids and soft semi‐solids due to its full contact with the sample (Frabetti et al. 2023). The MWS‐OECP is used to detect dielectric properties during sterilization processes in food industries, operating in a wide temperature range (−40°C to +200°C), and is ideal for low‐dielectric properties samples but may have reduced accuracy (Lau et al. 2020). However, air bubbles and uneven surfaces can cause inaccurate measurements, especially with heterogeneous samples (Keysight Technologies 2024a). It also requires a specific sample size and test platform and can form air bubbles, making it difficult to characterize large or heterogeneous foods and time‐consuming to determine permittivity. The OECP technique is the most commonly used method in MWS for food applications, as shown in Tables 4, 5, 6, 7, 8, 9.

**TABLE 4:** Detection of foreign bodies using MW sensing and imaging techniques.

Food product	Foreign bodies	Frequency (GHz)	MW Type	Measurement	Chemometrics/Algorithm	Experimental details	Findings	References
Dry foods (spaghetti, noodles, rice, wheat flour, and soy milk powder)	Metallic balls (steel, 2–5 mm diameter), copper wires (10–200 mm), and dielectric rods (plexiglass, 4–20 mm)	5.32–5.34	MWS	*S*_21_	– Self‐developed graphical user interface‐based software	– Cylindrical cavity resonator sensor where food passes through the cavity and metallic objects are detected by a shift in the resonant frequency – Perturbation theory – Fabrication cost approximately US$75	Detected of metallic balls, wires, and rods with low power consumption and low cost	Li et al. (2022)
Hazelnut‐cocoa cream in glass jar	Plastic fragments (2 mm)	9–11	MWI	*S*‐parameters, *ε*, *σ*	– MWT (linear and ill‐posed inverse problem) – TSVD algorithm – DBA for 3D	– Two antennas – Real‐time monitoring (in‐line)	– Monitored contaminants in packaged foods along production lines	Tobon Vasquez et al. (2019)
Hazelnut‐cocoa cream in glass jar	Plastic and glass fragments (2 mm)	1–20	MWI	*S*_21_, *ε*, *σ*	– MWT–TSVD algorithm	– Two (horn) antennas – Real‐time monitoring (in‐line) – 2D and 3D image	– Detected and localized contaminants in products using a classification framework	Tobon Vasquez et al. (2020)
Hazelnut‐cocoa cream in jars and water in bottle	PET plastic fragments (2 mm)	10	MWI	*ε*, *σ*, reflection coefficient	– DBA – TSVD algorithm – FEM solver (all for 3D image reconstructions)	– Dielectric properties – 3D image – In‐line inspection (conveyor belt)	– Detected 2 mm PET plastic contaminants in both cases of hazelnut cocoa cream jars and water bottles	Ricci et al. (2022)
Hazelnut chocospread	Plastic and stone	8–12	MWI	*S*_11_, *S*_12_	N.A.	– A horn antenna	Detect foreign bodies in homogeneous food products by using scattering parameters	Vp and Susan (2023)
Homogeneous foods (yogurt, oil, mayonnaise, milk, etc.)	Glass fragment (8 mm)	1–15	MWI	*S*_11_, *S*_22_, *S*_21_	– MWT–TSVD algorithm	– Parallel and orthogonal symmetry plane (PSP) and (OSP) based approaches – Two circularly loaded antipodal Vivaldi antennas	– Contaminants (small fragments of glass, plastic, or other materials) that might be present in the packaged homogeneous food were detected in circular plastic or glass jars	Zeni et al. (2023)
Oil	Plastic, glass, and wood fragments	10	MWS	*ε*, *σ*, reflection coefficient	– MLP	– Six antennas – In‐packaged food	– Real‐time contamination detection in packaged food from an industrial production line – Effective for detecting contaminants in low‐density plastics and other food and industrial sectors	Ricci et al. (2021a)
Safflower oil and hazelnut cocoa cream in jars	Metal sphere (10 mm), glass (13/2 mm), plastic sphere (3 and 20 mm), triangular plastic (8/1 mm), and cap shape plastic (15/9 mm)	10	MWS	Dielectric properties	– PCA, SVM, MLP (Bayesian optimization)	– Six antennas – Speed of conveyor belt = 3 jars/s (1 jar/333 ms)	– Detected the presence of contaminants in packaged jars when the liquid has different dielectric properties than the foreign bodies using SVM and MLP classifiers with the latter implemented in FPGA for real‐time (3 ms) – SVM set accuracy 93.958%	Urbinati et al. (2020)
Water	Plastic fragments (low density 2.5 mm)	2.5	MWI	*ε*, *σ*, reflection coefficient	– DBA – TSVD algorithm – FEM solver (all for 3D image reconstructions)	– Printed circuit board antenna – UWB antenna design – 3D image	– Detected mm‐sized plastic sphere in common commercial bottle filled with water	Ricci et al. (2022)

*Note*: *S*_11_ and *S*_22_, reflection coefficient; *S*_12_ and *S*_21_, transmission coefficient; *ε*″, dielectric loss factor; *ε*, permittivity; *ε*′, dielectric constant; *σ*, conductivity.

Abbreviations: DBA, distorted‐Born approximation; FEM, finite element method; MLP, multilayer perceptron; MWI, microwave imaging; MWS, microwave sensing; MWT, microwave tomography; N.A., not assessed; PCA, principal component analysis; SVM, support vector machine; TSVD, truncated singular value decomposition; UWB, ultrawide band.

**TABLE 5:** Detection of microorganisms and their toxins using MW sensing techniques.

Food product	Detection	Frequency (GHz)	Measurements	Mode/Type	Chemometrics/Classification	Experimental details	Findings	References
Water	*Escherichia coli*	7–8	*ε*	MWS	N.A.	– The sensor is 3 x 3 array of microtiter plate – Copper cavity	– Detection of 1–2 CFU of *Escherichia coli* in water at 4–5 min	Oberoi et al. (2012)
Milk (raw goat's milk)	Total bacterial count	0.02–4.5	*ε*′, *ε*″	MWS	PCA, SPA, PLS‐DA, SVM, ELM algorithms	– OECP methods – *N* = 150 (77 hygiene‐qualified and 73 hygiene‐disqualified milk samples)	– SVM‐PCA was the best model for identification of hygiene disqualified milk on total bacteria with accuracy rate of 100% – Dielectric spectra have great potential in in‐situ or on‐line bacteria detection	Zhu et al. (2018)
Wheat	Aflatoxin B1	2.5–11.5	Transmission index	MWS	BOSS, linear (PLSR) and non‐linear models (SVM, ELM, RF), cross validation	– Free space (broadband microstrip antenna) methods – *N* = 120 wheat	– BOSS can generate highly targeted feature variables – BOSS‐SVM model best prediction (2.8 µg/kg RMSEP, 0.97 *R*_P_^2^, and 5.7 RPD)	Xu et al. (2022)
Wheat	Aflatoxin B1 and mildew degree	3.5–12.5	Transmission index	MWS	Fusion CNN (multi‐task learning model)	‐ Free space (broadband microstrip antenna) – *N* = 200	– Fusion CNN model demonstrated excellent recognition performance, reaching 100% for qualitative analysis of the degree of mildew – Same model demonstrated great prediction (2.0138 µg/kg RMSEP, 0.9807 *R*_P_^2^, and 7.28 RPD) of quantitative aflatoxin B1	Deng et al. (2023)

*Note*: *R*_P_^2^, coefficient of determination; *ε″*, dielectric loss factor; *ε′*, dielectric constant.

Abbreviations: BOSS, bootstrapping soft shrinkage; CFU, colony forming unit; CNN, convolutional neural network; ELM, extreme learning machine; MWS, microwave sensing; N.A., not assessed; OECP, open‐ended coaxial‐line probe; PCA, principal component analysis; PLS, partial least squares; PLS‐DA, partial least squares discriminant analysis; PLSR, partial least squares regression; RMSEP, root mean square error of prediction; RPD, relative prediction deviation; SPA, successive projection algorithm; SVM, support vector machine.

**TABLE 6:** Moisture content (MC) measurement MW sensing and imaging techniques.

Food product	Frequency (GHz)	Mode/Type	Measurements	Chemometrics/Classification	Experimental details	Findings	References
Bulgur wheat, durum wheat, and corn silage kernel	1–2.48	MWS	*S*_11_, *S*_21_	KNN, SVR, ANN	– Free‐space method (two horn antennas)	– Best results are obtained with KNN for durum wheat (*R* ^2^ = 0.99) and corn silage kernel (*R* ^2^ = 0.98) and with SVR method for bulgur wheat (*R* ^2^ = 0.89)	Yigit and Duysak (2022)
Bulgur wheat, durum wheat, and corn silage kernel	1–2.48	MWI	*S*_11_, *S*_21_	CNN	– Free‐space method (two horn antennas) – 2D spectrogram image – *N* = 22,780 image	– MAE = 0.0411, MSE = 0.0149, RMSE = 0.122, and MAPE = 0.0397 – Completed training and testing in 325 s, compared to the 72‐min time required for the least time‐consuming architecture	Yigit et al. (2024)
De‐oiled cake (soya) and animal feed (cattle feed)	4.5	MWS	Transmission coefficient (phase and amplitude)	Linearity/Regression	– Waveguide (for calibration) and free‐space methods (two horn antennas) – Conveyor belt	– Monitored MC (0%–12%) of animal feed	Bekal et al. (2020)
Forage (alfalfa)	0.3–18	MWS	*ε*′, *ε*″	ANN	– OECP – *N* = 137	– Measured MC 11.5%–73%, wet basis, and bulk densities ranging over 139–716 kg m^−3^ (RMSE = 1.09%) – Determined MC in about 12 s, excluding sample preparation time	Shrestha et al. (2017)
Grain	8–12	MWS	*ε*′, *S*_11_, *S*_21_	N.A.	– Frequency selective surface antenna – Antenna sandwiched between an array of square split ring resonators at the top plate and a ground plate at the bottom	– Measured MC 10%–25%	Javanbakht et al. (2021a)
Granular materials (red black, green, and soy beans, rice, peanut)	2.45	MWS	Transmission coefficient and attenuation	N.A.	– Free space methods (microstrip patch array antenna)	– Measured MC 0%–30%	Jiarasuwan et al. (2021)
In‐shell peanut kernels	5.8	MWS	*ε*′, *ε*″	N.A.	– Free space transmission technique (two microstrip patch antennas) – *N* = 406 pod samples	– Results showed a standard error of performance of 0.53%	Trabelsi et al. (2016)
In‐shell peanut kernels, grain (wheat, corn, and soybeans), seed	11.3 and 18	MWS	*ε*′, *ε*″	N.A.	– Free‐space‐transmission technique (two microstrip patch antennas)	– The official moisture meter had a standard error of performance of 0.87% MC, whereas the MW moisture meter had a 0.53% error compared to the standard oven‐drying method	Trabelsi and Nelson (2016)
Milk (bovine)	40	MWS	*ε*′, *ε*″	Linear regression, repeated random sub‐sampling CV	– Waveguide – *N* = 150	– Detection of water content in bovine milk – Decrement of the signal was due to reduction of the free water volume	Agranovich et al. (2016)
Peanut	2–3	MWS	*S*_11_, *S*_21_	XGBoost algorithm, CARS, CARS–CV, PLS–CARS	– Two Vivaldi broadband antenna – NanoVNA – UWB transmitting antenna	– Validation set: *R* ^2^ = 0.9990, RMS = 0.1064, RMSE = 0.3262, MAE = 0.1937 – An actual moisture content of 35.57% and a maximum predicted error of 1.52%	Ma et al. (2023)
Potato (vacuum‐ and freeze‐dried)	2.45	MWS	*ε*′, *ε*″	Regression analysis	– OECP with portable VNA – 2 probe EIS	– *ε*′ and *ε*″ increased with temperature increases at 15%–18% MCs but were more consistent with higher MCs as long as no gelatinization occurred – Developed predictive biphasic models concerning starch gelatinization effects on *ε*′ and *ε*″ values of dried potatoes at 2.45 GHz	Chee et al. (2014)
Red winter wheat	10–18	MWS	Transmission coefficient	ANN	– Free space method (two horn antennas) – *N* = 179	– Measured MC (10.6%–19.2%), with a mean absolute error of 0.135 (*R* ^2^ = 0.99)	Bartley et al. (1998)
Rice	10.5	MWS	*ε*′, *ε*″, *S*_21_	A third‐order polynomial regression model	– Free space method (two horn antennas) – Waveguide/coaxial adaptors	– Detected short‐grain rough rice’ MC (12%–26%) with – *R* ^2^ = 0.986, SEP = 0.52%, and bias = 0.07%	Kim et al. (2002)
Wheat	2.5–11.5	MWS	Transmission index	CARS, GA, SVR	– Free‐space method (microstrip antenna) – *N* = 100	– Detected quantitative moisture content of wheat – CARS–GA–SVR model was best prediction (0.9756 *R* ^2^ and 6.3234 RPD)	Bai et al. (2024)
Wheat, rough rice, barley	4	MWS	Attenuate, *S*‐parameters (*S*_11_, *S*_21_, *S*_12_, *S*_22_, and complex permittivity	N.A.	– Coaxial waveguide – UWB radar—novel portable	– Measured wet basis (1%–26%) (*R* ^2^ = 0.99)	Zhang et al. (2019)

*Note*: *R* ^2^, coefficient of determination; *S*_11_ and *S*_22_, reflection coefficient; *S*_12_ and *S*_21_, transmission coefficient; *ε″*, dielectric loss factor; *ε′*, dielectric constant.

Abbreviations: ANN, artificial neural network; CARS, competitive adaptive reweighted sampling; CNN, convolutional neural network; CV, cross‐validation; GA, genetic algorithm; KNN, *k*‐nearest neighbor; MAE, mean absolute error; MAPE, mean absolute percentage error; MC, moisture content; MSE, mean square error; MWI, microwave imaging; MWS, microwave sensing; N.A., not assessed; OECP, open‐ended coaxial‐line probe; PLS‐CARS, partial least squares‐CARS; RMS, root mean square; RMSE, root mean square error; RPD, relative prediction deviation; SEP, square error of prediction; SVR, support vector regression; UWB, ultrawide band; XGBoost, eXtreme Gradient Boosting.

**TABLE 7:** Adulteration detection using MW sensing and imaging techniques.

Food product	Adulterant	Frequency (GHz)	Technique	Measurements	Chemometrics	Experimental details	Findings	References
Honey	Water (0%–80%)	1–20	MWS	*ε*′, *ε*″, tan *δ*	N.A.	– OECP	– Decreased *ε*′ of pure honey and water‐honey mixture with increasing frequency over 1–20 GHz range at room temperature – *ε*′ of pure honey was lower than that of water – Influenced permittivity of honey due to water content – *ε*″ of honey‐water mixture continued to drop as water content increased	Yakubu et al. (2019)
Honey (jujube honey, yellow‐locust honey, and milk‐vetch honey)	Sucrose syrup	0.01–4.5	MWS	*ε*′, *ε*″	N.A.	– OECP	– Decreased *ε*′ of pure jujube, yellow‐locust, and milk‐vetch honey, sucrose syrup, and honey–sucrose syrup mixture with increasing frequency over 0.01–4.5 GHz range at room temperature – *ε*′ of pure sucrose syrup was smaller compared with that of pure honey at any frequency – No significant linear correlation between *ε*′ and sucrose content in honey–sucrose syrup mixture – Dielectric relaxation was detected in pure honey, pure sucrose syrup, and honey–sucrose syrup mixtures. The relaxation frequency of sucrose syrup was similar to that of pure honey – Significant and strong negative linear correlation (*R* ^2^ > 0.98) between *ε*″ and sucrose content around relaxation frequency in all honey–sucrose syrup mixtures	Guo et al. (2011)
Honey (jujube honey, yellow‐locust honey, and milk‐vetch honey)	Water (18%–42.6%)	0.01–4.5	MWS	*ε*′, *ε*″	N.A.	– OECP	– Decreased *ε*′ of pure honeys and honey solutions with increasing frequency range and increased *ε*′ with water content – *R* ^2^ > 0.995, between ε′ and total soluble solids content and water content at 0.65–0.96 GHz – The penetration depth of electromagnetic energy in honeys decreased with increased frequency	Guo et al. (2010)
Milk (cow)	Water	0.01–4.5	MWS	*ε*′, *ε*″, dissipation factor tan *δ*	N.A.	– OECP – Milk concentration from 70% to 100% stored during 36 h storage at 22°C and 144 h	– Decreased *ε*′ of milk with the increasing frequency – Raw milk had lowest *ε*′ when the frequency was higher than about 20 MHz and had the highest loss factor over the detected frequency range when compared with diluted milk – Increased penetration depth with decreasing frequency, water content, and storage time, which was large enough to detect dielectric properties changes in milk samples	Guo et al. (2010)
Milk (fresh raw, cow)	Water adulteration duration 0, 18, and 36 h at 22°C	0.01–4.5	MWS	*ε*′, *ε*″, or tan *δ*, conductivity and penetration depth	N.A.	– OECP	– The raw milk had the lowest *ε*′ when the frequency was >20 MHz and had the highest *ε*′ or tan *δ* at each frequency when compared with diluted milk – Increased penetration depth with decreasing frequency, water content, and storage time, which was large enough to detect dielectric properties changes in milk samples – *ε*″ can be an indicator in predicting milk concentration and freshness – Decreased *ε*′ of milk with the increasing frequency	Jitendra Murthy et al. (2017)
Milk (whole milk or defatted milk (skim milk))	Water and urea adulteration	0.1–20 GHz	MWS	ε′, ε″	N.A.	– OECP	– Detect the existence of polar adulteration like urea and water in milk – DRPs of milk adulterated with water or urea significantly differed from those of pure milk. These findings suggest that DRPs are highly responsive to component information present in cow's milk.	Zhao et al. (2019)
Wheat flour	Talcum powder	2.5–11.5 GHz	MWS	Transmission index	BOSS, MEF‐LASSO, CARS, XGBoost classification and regression models, and NIOA, OOA, CPO, SSA	– Two antennas	– SSA‐XGBoost classification model highest prediction accuracy (100%) – RMSE = 1.76 – *R* ^2^ = 0.98 – RPD = 8.66	Xu et al. (2024)

*Note*: *R* ^2^, coefficient of determination; *ε′*, dielectric constant; *ε″*, dielectric loss factor.

Abbreviations: BOSS, bootstrapping soft shrinkage; CARS, competitive adaptive reweighted sampling; CPO, crested porcupine optimizer; MEF‐LASSO, multiple feature spaces ensemble with least absolute shrinkage and selection operator; MWS, microwave sensing; N.A., not assessed; NIOA, nature‐inspired optimization algorithms; OECP, open‐ended coaxial‐line probe; OOA, osprey optimization algorithm; RMSE, root mean square error; RPD, relative prediction deviation; SSA, sparrow search algorithm; XGBoost, eXtreme Gradient Boosting.

**TABLE 8:** Application of MW sensing and imaging techniques in food quality control.

Food product	Detection	Frequency (GHz)	Mode/Type	Measurements	Chemometrics	Experimental details	Findings	References
Egg	Freshness	0.9–1.7	MWS	*S*_11_, *S*_21_	PLS, ANN regression (for predicting egg quality), SIMCA, and ANN classification (classify the eggs)	– Waveguide	– The best predictive models obtained from ANN analysis were where the yolk coefficient, air cell height, thick albumen height, Haugh unit, and albumen pH could be predicted with the residual predictive deviation (RPD) values of 3.500, 3.000, 2.411, 2.033, and 1.829, respectively – To classify the eggs according to their storage time, both SIMCA and ANN analyses resulted in the total accuracy of 100% when *S*_11_ spectra were used as the input – SIMCA classified the eggs based on storage time	Akbarzadeh et al. (2019)
Egg	Thin albumen, thick albumen, and yolk	7–13	MWI	*ε*″, *σ*, *S*_11_, *S*_12_, *S*_21_, *S*_22_	N.A.	– Transmitting waveguide antenna	– Thicknesses and shapes can be detected, enabling the evaluation of egg health	Tai et al. (2020)
Frozen sucrose solution	Moisture content	0.003–3	MWS	Intensity (mV)	N.A.	– OEC resonator – During freezing process—concentration and temperatures	– Successfully monitored freezing process	Kono et al. (2021)
Fruits (apple, avocado, dragon fruit, guava, and mango)	Identification and ripeness grading	0.7–1	MWS	*S*_11_, *S*_21_	KNN and neural network	– Two antennas – NanoVNA	Classification accuracy of the neural network model was higher than KNN with 98.75% and 99.75% KNN was seen to be more effective in classifying ripeness with 98.4% as compared to 96.6% for neural network	Tran et al. (2023)
Lemons and grapefruits	Seeds (inside)	1–10	MWI	Dielectric properties, *S*_21_	N.A.	– Radar algorithm—Huygens principle‐based algorithm – 2D	– Indicated the capability of the algorithm to both detect seeds inside the fruits and distinguish between seeded and seedless samples	Ghavami et al. (2019)
Liquid solutions (sodium chloride, citric acids, saccharose) and liquid food (milk, egg products, fruit juice)	Concentration (solutions and egg products), Brix (fruit juice, temperature, and concentration (milk)	<3	MWS	Real and imaginary parts of *S*_11_	PLSR, PCA	– OECP – NanoVNA	– *R* ^2^ = 0.933–0.998	Iaccheri et al. (2022)
Meat (chicken, salmon, and trout)	Transglutaminase	0.433, 0.915, and 1.8	MWS	*ε*′, *ε*″	N.A.	– OECP	– ε′ was similar between the transglutaminase‐treated chicken muscle and the control, but the *ε*″ was higher for the transglutaminase‐treated tissue	Basaran et al. (2010)
Meat (lamb, chicken, beef, and pork)	Water‐holding capacity	4.6–5.6	MWS	*S*_11_, *S*_21_	N.A.	– Resonator (cavity‐rectangular)	– Real‐time measurement of water‐holding capacity of meat during 24 h	Abdullah et al. (2014)
Meat (pork)	Quality defects	0.5–10	MWS	*ε*′, *ε*″	N.A.	– OECP – Quality defects in porcine muscle during postmortem period	– Evolution of dielectric spectra during meat aging showed important variations only in dark, firm, and dry (DFD) meats, whereas in pale, soft, and exudative (PSE) and red, firm, and non‐exudative (RFN) meats the variations were observed only at some punctual frequencies of the spectrum during the 24 h after slaughter	Castro‐Giráldez et al. (2010)
Milk (cow) (raw)	Freshness, concentration, and deterioration	0.01–4.5	MWS	*ε*′, *ε*″	N.A.	– OECP – Deterioration during 36 h storage at 22°C and 144 h at 5°C	– *R* ^2^ = 0.995 for milk concentration	Guo et al. (2010)
Milk (UHT whole, low fat, and skim milk)	Deterioration	1–20	MWS	Complex permittivity	N.A.	– OECP – Deterioration at 17–20°C in a period of 2 weeks	– Varied spectra with spoilage, but analysis is complicated by concerns of physical (phase separation) in addition to chemical changes	Nunes et al. (2006)
Pears (Korla Fragrant)	Soluble solid content and hardness	0.1–26.5	MWS	*ε*′, *ε*″	PLSR, SVR, PSO–LSSVR	– OECP	– PLSR best prediction method – *R* = 0.77 and MSE = 0.073 (for *ε*′ and hardness) – *R* = 0.91 and MSE = 0.087 (for *ε*″ and soluble solid content)	Tang et al. (2024)
Potato	Air gaps (inside) detection and localization	1–8	MWI	—	Huygen‐based radar algorithm	– MW radar imaging prototype – Spear shape antennas (3 transmitting and 13 receiving)	– Experimental prototype can detect potato defects	Ghavami et al. (2021)
Potato and apple	Freshness (fresh or stale)	4.9–7.05	MWS	*S*_11_, *S*_21_, *μ*, and *ε*′	Nicolson–Ross–Weir (NRW) algorithm (non‐iterative method)	Rectangular waveguides	– Freshness or staleness of food products affects the electrical properties	Ates et al. (2017)
Syrup	Brix	2.45	MWS	*ε*′, *ε*″	N.A.	– Open‐coaxial resonator sensor (resonant cavity) – *N* = 37	– ‐Syrup 55–90 °Bx values increase with the increase in Brix – A reliable quadratic and quartic relationship between quality factors and real and imaginary permittivity, respectively	
Tomato paste	Soluble solids content, titratable acidity, and consistency	0.136–2.69	MWS	Signal (transmitted) amplitude	PCA, PLS, iPLS	– Parallel plates (rectangular section) acts as a waveguide – Inline sensor – Analysis in dilution and evaporation conditions	– iPLS—Bostwick consistency *R* > 0.92 and RMSECV <0.7 cm – PLS—quantification of SSC and TA—*R* > 0.95 – Bostwick consistency *R* > 0.8	Zhang et al. (2014)
Walnut	Freshness (empty, fresh, and rancid)	7–12	MWI	*S*_11_, *S*_22_	ANN	– Two UWB Vivaldi antennas – *N* = 180	– Minimum error rates for glued‐unshelled, healthy‐unshelled, and rancid walnut using reflection coefficients were 7.33%, 16.92%, and 7.92%, respectively	Kizilay et al. (2023)
Watermelon	Maturity (ripe, barely ripe, and unripe)	2–8	MWI	*S*_21_	DAS beamforming algorithm and MERIT	– Circular array with 10 coplanar Vivaldi antennas	– Detected internal characteristics of watermelon, including water and sugar concentration and distribution, with high permittivity categorized by red heat signatures	Garvin et al. (2023)
Watermelon	Ripeness level	2–8	MWI	—	DAS beamforming and CNN	– 10 UWB Vivaldi antennas	– 86% accuracy (in threefold cross validation)	Choffin et al. (2025)

*Note*: *R* ^2^, coefficient of determination; *ε′*, dielectric constant; *ε″*, dielectric loss factor; *μ*, permeability; *S*_11_ and *S*_22_, reflection coefficient; *S*_12_ and *S*_21_, transmission coefficient.

Abbreviations: ANN, artificial neural network; DAS, delay‐and‐sum; iPLS, interval PLS; KNN, *k*‐nearest neighbor; MSE, mean square error; MWI, microwave imaging; MERIT, microwave radar‐based imaging toolbox; MWS, microwave sensing; N.A., not assessed; OECP, open‐ended coaxial‐line probe; PLS, partial least square; PLSR, partial least squares regression; PSO‐LSSVR, particle swarm optimization–least squares support vector regression; PCA, principal component analysis; RPD, residual predictive deviation; SIMCA, soft independent modeling by class analogy; SSC, soluble solid content; SVR, support vector regression; TA, titratable acidity; UWB, ultrawide band.

**TABLE 9:** Detection of the composition of foods using MW sensing.

Food product	Detection	Frequency (GHz)	Mode/Type	Measurements	Chemometrics/Classification	Experimental details	Findings	References
Fruit juices	Carbohydrates and water content	1.6–2.7	Waveguide	Gain and phase acquired waveforms	PLSR and CV	– Qualitative estimation – Online	– PLSR (*R* ^2^ = 0.990)	Ragni et al. (2017)
Milk	Fat						– PLSR (*R* ^2^ = 0.991)	
Water	Fructose, ethanol, sodium chloride						– PLSR (*R* ^2^ = 0.999)	
Meat (dry‐cured ham)	Water activity, water, and salt contents	2–6	Resonator (cavity‐rectangular)	Amplitude of *S*_11_ and *S*_21_	N.A.	– —	– *R* ^2^ = 0.76–0.98 (correlation between amplitude and quality parameters)	Bjarnadottir et al. (2015)
Meat (ground beef)	Fat content	0.2–20	OECP	*ɛ*′, *ɛ*″, loss tangent	MSC, SNV, PCA, PLSR	– —	– *R* ^2^ = 0.87 and RMSEP = 2.71% (w/w) at 12.9–20 GHz (for *ɛ*′)	Zhao et al. (2016)
Milk (whole, semi‐skimmed, and skimmed)	Fat and protein content	0.01–15	Resonator (cavity)	*S*_11_, *S*_21_	N.A.	– —	– *R* ^2^ = 0.98 (for fat) (linear regression with correlation) – *R* ^2^ = 0.949 (for protein)	Joshi et al. (2017)
Milk (whole and skim)	Lactose content	0.02–4.5	OECP	*ε*′, *ε*″	N.A.	– —	– *ε*′ decreased as increasing frequency – Whole milk had lower dielectric properties than skim milk – *ε*′ had weak positive linear relationship with lactose content for whole milk, but had negative linear relationship for skim milk – *ε*″ had very good negative linear relationship with lactose content <1 GHz and had good positive linear relationship >2.3 GHz	Liu et al. (2018)
Milk (skim milk)	Solids‐not‐fat content						– *ε*′ of skim milk decreased as frequency increased, and *ε*″ changed from decreasing to increasing with minimums at about 2 GHz – *ε*′ and solids‐not‐fat content *R* ^2^ > 0.940 below 50 MHz and *R* ^2^ = −0.990 above 150 MHz – *ε*″ and solids‐not‐fat content *R* ^2^ > 0.970 at all frequencies – Penetration depth of EM wave in milk decreased with increased frequency or solids‐not‐fat content	
Oil (edible soybean)	Pb	2–12	N.A.	Transmission coefficients	CNN and residual attention CNN	– Pb concentrations of 50–0.03 mg/kg	– RMSE = 3.1654 mg/kg – *R* ^2^ = 0.9605 – RPD = 5.0479	Deng et al. (2024)
Solution of proteins and enzyme	Enzyme activity	5.5–7.5	OECP (fork type)	*ε*′	N.A.	– 10:1 Protein (egg white, egg yolk, plant protein, insulin): papain enzyme	– Decrease in *ε*′ by addition of papain enzyme to protein	Urvashi et al. (2019)

*Note*: *ε′*, dielectric constant; *ε″*, dielectric loss factor; *R* ^2^, coefficient of determination; *S*_11_ and *S*_22_, reflection coefficient; *S*_12_ and *S*_21_, transmission coefficient.

Abbreviations: CNN, convolutional neural network; CV, cross validation; EM, electromagnetic; MSC, multiplicative scatter correction; N.A., not assessed; OECP, open‐ended coaxial‐line probe; PCA, principal component analysis; PLSR, partial least squares regression; RMSE, root mean square error; RMSEP, root mean square error of prediction; RPD, relative prediction deviation; SNV, standard normal variate.

#### 2.2.2 Free Space Method

Free space methods use antennas (e.g., horn and Vivaldi) to focus MW radiation at or through a material slab, as shown in Figure 3. The system mainly consists of VNA, two antennas or an antenna array, FUT, and software (Chandra et al. 2015). The technique is based on the transmission line theory for material characterization measurement for transmission (*S*_21_) and reflection coefficients (*S*_11_). Like the OECP method, this method is one of the most widely used methods in food applications, as MWS. But the free‐space method is a non‐contacting and non‐destructive method suitable for high temperatures and mm‐wave frequencies (30–300 GHz/1 cm–1 mm) (Keysight Technologies 2024b). Moreover, it can measure all types of food samples, including liquid, solid, semi‐solid, heterogeneous, homogeneous, packaged, and unpackaged. For instance, MC detection of grains (wheat and silage kernel) was evaluated using the MWS free‐space method with two horn antennas based on transmission line theory (Yigit and Duysak 2022). Moreover, hazelnut‐cocoa cream in a glass jar (Tobon Vasquez et al. 2019), oil (Ricci et al.), in‐shell peanut kernels (Trabelsi et al. 2016), potato, rice, and so forth were analyzed with MWS free space methods, as shown in Tables 4, 5, 6, 7, 8, 9.

#### 2.2.3 Resonator (Cavity or Planar)

The resonator technique measures the dielectric properties of a food sample by analyzing the shift of resonant frequency (*f*) and the change in cavity quality (*Q*) factor. It calculates permittivity by inserting the food sample and computing its dielectric properties using the frequency, the *Q*‐factor, and the sample volume with a VNA (Peng et al. 2014). Resonant cavities are high *Q* structures with specific frequencies influenced by food samples, allowing permittivity calculation based on center frequency and *Q* factor (Peng et al. 2014; Lewis et al. 2024). The MWS technique can be divided into two types: Those achieved by dielectric samples and those promoted by cavity metallic walls. It is based on the shift of resonant frequency and *Q* factor due to a small sample (Peng et al. 2014; Lewis et al. 2024). It is reliable for dielectric measurements of various foods over various frequencies, temperatures, and MCs. The system uses simple and inexpensive materials for dielectric properties measurements. Moreover, other advantages are that it provides easy preparation, fast measurement, and accurate results (Frabetti et al. 2023). For instance, the MWS cavity perturbation method accurately determined the dielectric properties of oils (mustard, olive, sesame, and canola) using a two‐port VNA connected to each end of the cavity resonator (Praveen Kumar et al. 2019). The cavity perturbation method is particularly sensitive and requires a small sample because the maximum change in resonant frequency occurs with a slight perturbation at the peak intensity of the cavity mode (Gioia et al. 2018). This MWS method was successfully used for measuring water content, density, and concentration of foods. In addition to water activity and water content, the salt content of dry‐cured ham was detected using a rectangular cavity resonator using reflected and transmitted signals (Bjarnadottir et al. 2015). Liu et al. (2022) measured the dielectric properties of syrup and its Brix using a resonant cavity at 1.9 GHz. Besides, the glucose concentration of water‐glucose solutions was measured by a hyper‐sensitive MW sensor based on a split ring resonator using the dielectric properties at 1.9 GHz (Zidane et al. 2021). MW has low penetration in metals, which act as reflectors and absorb little EM energy (Li et al. 2022). Li et al. (2022) utilized cylindrical cavity resonators to detect foreign bodies in dry foods, revealing that frequency variation is directly linked to object volume.

This MWS technique has several limitations, for example, it primarily provides dielectric data at a single frequency, ranging from 1 to 50 GHz (Gioia et al. 2018). First, the sample size is quite small, which can interfere with the reproducibility of the tests. Additionally, different cavities have specific size and shape requirements, limiting the method's versatility. Although the equipment for this technique is relatively affordable, the preparation of samples is complex and usually requires precise cutting and molding of the food items, and measuring other solid food samples can be problematic. Thus, this method works well for liquids, semi‐liquids, and food powders like wheat, rice, paprika, and milk powder (Frabetti et al. 2023; Xu et al. 2024). Dry foods absorb less energy, allowing for deeper signal penetration due to their low dielectric loss factor value (Li et al. 2022). Issues such as air pockets forming within the sample or between the sample and the cavity, fluid loss, and increased density from pressing the samples into the cavity can all affect the measurement accuracy (Gioia et al. 2018). Moreover, MW energy is readily absorbed by moist samples, which can lead to the degradation of resonance and reduce effectiveness with high‐moisture foods.

#### 2.2.4 Waveguide Method

A waveguide is a type of transmission line consisting of a hollow metal tube that guides waves. These waveguides in Figure 3 are connected to a two‐port VNA to measure the perturbation of EM fields due to the food sample. That is, it is typically connected to a network analyzer, transmits MW to the sample, and measures *S*‐parameters in both reflection and transmission modes and dielectric properties (*ε*′ and *ε*″) (Sharma 2011). The food sample is inserted between the transmitter and receiver antennas inside the waveguide. Another method involves cutting the food sample into the same size as the waveguides and sandwiching it between two rectangular waveguides. All types of food samples can be measured with MWS waveguide methods to detect food quality and safety, as shown in Tables 6, 8, and 9. For instance, the freshness of the egg was measured by the waveguide transmission line method using reflection and transmission measurement modes at 1.12–1.7 GHz (Akbarzadeh et al. 2019). Two waveguides were used to accurately measure the water and MC of bovine milk, as reported by Agranovich et al. (2016).

#### 2.2.5 PP Method

The PP is an MWS method for measuring dielectric properties by sandwiching a flat sample or a thin sheet of food samples between two electrodes to form a capacitor (Lau et al. 2020). The method operates most effectively for precise, low‐frequency measurements of thin sheets or liquids. A typical measurement system using the PP method consists of an inductance (L), capacitance (C), and resistance (R) meter or impedance analyzer to calculate the sample's dielectric constant and loss factor based on its parallel capacitance and resistance values (Lau et al. 2020). It can be applied to frequency ranges covering up to 1 GHz (Keysight Technologies 2024b). The PP mainly works in radiofrequency and small bands of MW regions. Thus, there are few studies related to food applications. For instance, the OECP and PP methods measured dielectric properties of low‐moisture foods such as ground black pepper, all‐purpose bleached wheat flour, non‐fat milk powder, and whole milk powder at 5 MHz–3 GHz and 5–30 MHz, respectively (Lau et al. 2020). Although the OECP is more suitable for MW frequencies, the PP is better for radiofrequency and low dielectric properties. Moreover, soluble solid content, titratable acidity (TA), and consistency in dilution and evaporation of tomato paste were measured using MWS–PP (at 0.136–2.69 GHz) with chemometric methods (principal component analysis [PCA], partial least square regression, etc.) (Zhang et al. 2014), and the results showed an accurate prediction (*R* > 0.92) for three parameters tested.

### 2.3 Microwave Imaging

Food imaging is an important tool for sample visualization for food safety and quality in a non‐invasive way. Several imaging techniques, including X‐ray imaging (Kotwaliwale et al. 2014), magnetic resonance imaging (Kirtil and Oztop 2015), hyperspectral imaging (Ma et al. 2019), Raman, fluorescence, and laser backscattering imaging (Hussain et al. 2018), and terahertz imaging (Afsah‐Hejri et al. 2020), have been used in food applications. Recently, MWI techniques are in the early development stage for food applications, as they are being researched and explored in the food industry. MWI has excellent penetration characteristics (1–30 cm) (Hussain et al. 2018). Moreover, MWI has been proposed for online testing of food samples, the detection of possible defects, and measurements of physical quantities on conveyed products (Noghanian et al. 2014). MWI can detect contaminants not visible to x‐ray systems, such as plastic or glass fragments in food products (Tang et al. 2022). For instance, bruises on different apples were detected to measure the relative dielectric constants of apples using multi‐resolution MWI, thereby distinguishing good visualization apples from bruised ones (Lu et al. 2018). Tobon Vasquez et al. (2020) detected small contaminants (e.g., a few mm in size of plastic or glass chips) in packaged food during production line movement with a novel foreign body detection MWI device using dielectric properties. Another study showed that the chemical composition (water, sugar content, etc.) of fruit, maturity, and defects of agricultural products, mold, or potential foreign organisms and contaminants were detected using MWI, ensuring real‐time monitoring (Ghavami et al. 2021; Tang et al. 2022). MWI techniques can be categorized into radar‐based imaging (RI) and MW tomography (MWT), with MWT being a common method for food analysis.

#### 2.3.1 Microwave Tomography

MWT is a quantitative algorithm used to map the distribution of food materials by solving inverse scattering problems (Rubæk and Mohr 2016). Tomographic imaging for food applications uses algorithms to reconstruct electrical properties, helping to identify regions of interest based on known electrical properties. This method involves both linear and nonlinear approaches. In MWT, a nonlinear inverse scattering problem is solved to accurately reconstruct the dielectric properties of a target sample, offering a precise depiction of its internal structure (Noghanian et al. 2014; Rubæk and Mohr 2016). In contrast, RI offers a simpler computational approach. However, MWT's precision makes it valuable for real‐time contaminant detection and food safety assessment.

#### 2.3.2 Radar‐Based Imaging

Qualitative algorithms, which use radar‐like techniques, focus on identifying food materials and detecting adulteration or contamination without the need for computationally intensive iterative algorithms (Chandra et al. 2015). The transmitter antenna (TX) emits EM waves, creating scattered and backscattered signals from the object of interest (OI) (Noghanian et al. 2014). It detects contrasts in the imaging medium but does not provide direct values for dielectric properties. For instance, Ghavami et al. (2019) explored the application of a newly developed MW radar algorithm for fruit imaging. They analyzed variations in the dielectric properties of fruits to detect internal structures using custom‐built hardware and a Huygens principle‐based reconstruction algorithm. Their experimental results on lemons and grapefruits demonstrate the algorithm's ability to identify seeds and differentiate between seeded and seedless samples, highlighting its potential for non‐invasive food quality assessment.

Synthetic aperture radar (SAR) reconstructs contrast based on scattered fields from different locations, identifying strong scatterers rather than providing a dielectric profile (Noghanian et al. 2014). Radar technologies like confocal MWI, beamforming, and ultra‐wideband (UWB) enhance resolution, though confocal MWI is limited by frequency‐dependent effects and noise. MWI via space‐time beamforming, which spatially focuses backscattered signals, is proposed as a more effective method (Chandra et al. 2015).

### 2.4 Data Analysis

Machine learning (ML) is a powerful technology that mimics human learning by extracting patterns from data. In the context of MWS and MWI, ML has been instrumental in identifying critical features that enhance food quality and safety detection and assessment. The process begins with data preprocessing, which involves signal correction and normalization (NM) to improve data reliability. Following this, feature selection techniques help identify the most relevant MW sensor parameters. Finally, ML or chemometric methods are applied to develop predictive models, improving the accuracy and efficiency of food contamination detection, adulteration analysis, and quality monitoring. Overall, ML and classifiers trained on raw MW signals and images can eliminate the need for complex imaging algorithms, enabling faster, real‐time detection of contaminants and adulterants (Urbinati et al. 2020). This approach enhances efficiency and simplifies processing, making MWI (as well as MWS) more practical for food quality assessment.

#### 2.4.1 Data Preprocessing

Various preprocessing techniques are employed to remove irrelevant information caused by sample heterogeneity, air bubbles, and mechanical motion in MWS. Common methods include NM, standard normal variate (SNV), multiple scatter correction (MSC), Savitzky–Golay (SG) filtering, baseline offset correction (BOC), and first/second derivatives, which help correct the scatter effects, eliminate baseline deviation, and improve regression model (Zhu et al. 2018; Akbarzadeh et al. 2019; Halim et al. 2022). For instance, Zhu et al. (2018) tested SG, SNV, and their combination on milk dielectric spectra, finding that SG + SNV yielded the best model performance and noise reduction. Moreover, Akbarzadeh et al. (2019) evaluated MSC, NM, SNV, BOC, and first/second derivatives for egg freshness detection, concluding that second derivatives were the most effective preprocessing method. Xu et al. (2022) applied least squares (LS) filtering (window size = 19 and polynomial order = 2) to preprocess wheat transmission index data before multivariate MW analysis. These findings highlight the importance of optimized preprocessing techniques for enhancing the accuracy and interpretability of MW‐based food quality assessment.

#### 2.4.2 Feature Selection

Feature selection is a key preprocessing step in chemometrics that improves data visualization, prevents over‐fitting, and reduces training time by selecting the most relevant features (Soltani Firouz et al. 2022). It enhances predictive model efficiency by minimizing input variables, lowering computational costs, and distinguishing class‐correlated data (Halim et al. 2022). Statistical feature selection techniques quickly assess relationships between input and target variables, selecting the most informative ones. Xu et al. (2022) applied bootstrapping soft shrinkage (BOSS) to optimize feature selection for aflatoxin B1 detection in wheat, combining variables and extracting relevant information through model population analysis. Moreover, Soltani Firouz et al. (2022) used the correlation‐based feature selection (CFS) on dielectric spectra to eliminate irrelevant and refine the dataset for improved analysis. These methods enhance the accuracy and efficiency of MW‐based methods and spectral analysis in food quality assessment.

#### 2.4.3 Machine Learning

MW systems combined with ML provide an effective approach to food quality and safety assessment. After data preprocessing, feature extraction from MW signals enables the application of regression and classification algorithms, improving predictive accuracy and reliability. Regression models include linear approaches such as multiple linear regression (MLR), principal component regression (PCR), and partial least squares regression (PLSR), as well as non‐linear methods like LS‐support vector machines (LS‐SVM) and artificial neural networks (ANN) (Halim et al. 2022; Soltani Firouz et al. 2022). Classification models can be either supervised, including linear discriminant analysis (LDA), partial least squares discriminant analysis (PLS‐DA), *k*‐nearest neighbor (KNN), soft independent modeling of class analogy (SIMCA), and SVM, or unsupervised, such as PCA and hierarchical clustering analysis (HCA) (Zhu et al. 2018; Akbarzadeh et al. 2019). All chemometric methods were used for data evaluation after MW‐based detection, as shown in Tables 4, 5, 6, 7, 8, 9.

PCA is widely used to reduce data dimensionality while retaining key information, improving prediction accuracy. It has been applied to detect bruises in apples using MWS by identifying characteristic frequency changes (Lu et al. 2018). Moreover, PLSR extracts latent variables to predict food quality parameters and has been employed in MWS for quantitative modeling (Nicolaï et al. 2007). SVM, a robust supervised classification algorithm, constructs an optimal hyperplane to differentiate between food classes with minimal error, ensuring reliable classification (Byvatov and Schneider 2003; Raghavendra and Deka 2014). ANN, a powerful non‐linear regression and classification tool, has been successfully applied in food quality studies. For example, ANN models demonstrated high accuracy in evaluating egg freshness using a waveguide‐equipped MWS by analyzing *S*‐parameters and achieved 100% accuracy in classifying eggs based on storage time, showcasing their effectiveness for food monitoring (Akbarzadeh et al. 2019).

Besides, MWS, combined with ML models, has been extensively used for detecting food adulteration and contamination. ANN classifiers achieved 100% accuracy in distinguishing authentic and adulterated sesame oil, demonstrating their robustness in quantitative adulteration analysis (Soltani Firouz et al. 2022). Similarly, MW‐based detection (free‐space) of aflatoxin B1 in wheat has been conducted using both linear (PLS) and non‐linear models (SVM, extreme learning machine (ELM), and random forest), providing accurate concentration measurements (Xu et al. 2022). Additionally, MWS has been integrated with ML for real‐time contaminant detection in food packaging. Researchers successfully identified plastic and glass contaminants in cocoa hazelnuts using antenna arrays for *S*‐parameters analysis. For instance, ML classifiers, including SVM and multilayer perceptron (MLP), were trained to classify contaminants, with MLP implemented in FPGA hardware for real‐time execution, showcasing the industrial potential of MWS (Urbinati et al. 2020).

## 3 Food Applications of MWS and Imaging

Traditional mechanical and chemical methods (HPLC, LC, GC, and LC/HPLC/GC coupled with mass and atomic absorption spectrometry) are destructive, time‐consuming, and inadequate for modern needs. Moreover, these methods require sample purification, well‐trained experts, and large‐scale instruments, their high cost and off‐line, thereby making them unsuitable for real‐time food quality assurance and safety monitoring. The increasing demand for quality assurance in food production necessitates sophisticated analytical methods for objective quality control. Non‐invasive and non‐destructive food detection technologies have gained significant concern due to their ability to provide accurate results and to be rapid, easy, and cost‐effective methods. Current non‐destructive spectroscopic and imaging techniques have been extensively investigated, including x‐ray (Haff and Toyofuku 2008; Kotwaliwale et al. 2014), impedance spectroscopy (Nakonieczna et al. 2016), magnetic resonance imaging (MRI) (Ebrahimnejad et al. 2018), near‐infrared (NIR) spectroscopy and imaging (Fu and Ying 2016; Beć et al. 2022), Raman spectroscopy (Sun et al. 2022), hyperspectral imaging (Ma et al. 2019), and terahertz (THz) spectroscopy and imaging (Afsah‐Hejri et al. 2020) due to their rapid and easy food quality and safety control. These methods have been used for qualitative and quantitative analysis, such as detecting chemical constituents of food, food fraud, food additives, and contaminants (e.g., heavy metals, plastics, and pesticides).

Similarly, MW sensing (MWS) and imaging (MWI) are non‐destructive methods that provide valuable information about food quality and safety. For this purpose, these systems use dielectric properties (mainly permittivity (ε′)), *S*‐parameters, and impedance data. Then, from the data, the information can be derived on the food sample's structural, physical, and chemical features. MW spectral data results can be analyzed with ML or chemometric methods. In the past decade, ML has been commonly used to reduce variables and facilitate the analysis of large amounts of data.

This section reviews MWS and MWI technologies for the detection and quantification of contaminants in food. These include foreign bodies, microorganisms and their toxins, and food adulteration (e.g., honey, milk). Additionally, the applications of MW techniques in MC measurement, food quality control, and composition analysis were explored.

### 3.1 Contamination Detection

Contamination in food products is a growing global concern, posing significant food safety challenges due to harmful substances that can cause adverse health effects (Hussain 2016). Food contaminants are classified into three contamination types: biological, physical, and chemical. These are mostly microorganisms (bacteria, viruses, etc.), foreign bodies (plastic, metal, glass, wood, etc.), pesticides, insecticides, heavy metals, antibiotics, and so on. This section explores the detection of foreign bodies, microorganisms, and their toxins using MWS and MWI techniques.

#### 3.1.1 Foreign Body (Plastic, Metal, Glass, Etc.)

Foreign bodies, such as plastic, glass, metal, wires, and stone, might contaminate food products during manufacturing, handling, and packaging. Detecting metal contaminants in food is easier than nonmetal ones, and x‐ray imaging is mainly used for this purpose (Haff and Toyofuku 2008). However, it is unsuitable for low‐density contaminants in optically opaque food products. It can pose a significant risk to workers near the production chain, as they may accidentally be exposed to radiation. X‐ray and nuclear magnetic resonance (NMR) instruments have high cost and utilize high power. Moreover, infrared (IR) is limited by the short penetration depth in the food sample.

In contrast, MWI and MWS are safe for use and can detect foreign bodies, not only metal and plastic fragments but also stone, glass, low‐density plastic, and wire at a range of 1–20 GHz (Table 4). On the other hand, foreign bodies can be detected in‐line/real‐time or online using the antenna(s). For instance, Zeni et al. (2023) used MWI in‐line monitoring systems for detecting foreign bodies in homogeneous food (such as yogurt, oil, mayonnaise, and milk) contained in a plastic/glass holder of circular shape. They collected *S*‐parameters such as the reflection coefficient (*S*_11_ and *S*_22_) and transmission coefficient (*S*_21_) with Vivaldi antenna at 1–15 GHz. The glass fragments of 8 mm in the jar were detected by MWI. Vp and Susan (2023) also measured *S*‐parameters (*S*_11_ and *S*_12_) of uncontaminated and plastic‐contaminated food products. They found that input reflection coefficient *S*_11_ is most suitable for measurements due to its higher sensitivity compared to transmission coefficient *S*_12_. The reflection coefficient *S*_11_ for uncontaminated food products, in comparison to those of plastic and stone‐contaminated choco spread, showed that contaminants increase the reflection coefficient *S*_11_. Thus, uncontaminated food products can be easily distinguished from contaminated products by comparing the measured scattering parameters (*S*_11_). Additionally, *S*_11_ is measured by adjusting the distance between the food product and the antenna. However, changing the distance has no impact on the value of *S*_11_.

Li et al. (2022) showed that metallic balls (steel, 2–5 mm diameter), copper wires (10–200 mm), and dielectric rods (plexiglass, 4–20 mm) were detected in dry foods such as spaghetti, noodles, rice, wheat flour, and soy milk powder with transmission coefficient (*S*_21_) at 5.35 GHz MW resonator sensor with low power consumption and low cost. Other studies demonstrated that 2‐mm plastic and glass fragments in hazelnut‐cocoa cream were detected using MWI at 1–20 GHz using MWT with two antennas and a truncated singular value decomposition (TSVD) algorithm in‐line inspection (Tobon Vasquez et al. 2019, 2020). Ricci et al. (2022) developed their UWB antenna at 2.5 GHz to detect plastic contamination of water‐based food products. They detected a 2.5 mm low‐density plastic fragment in commercial bottle‐filled water with 6 UWB antennas at in‐line inspection. Moreover, they suggested that MWI can be used to monitor contamination in water‐based food products and should demonstrate its potential for in‐line production without halting processes or reducing speed. The same authors detected 2 mm PET plastic contaminant in hazelnut‐cocoa cream jars and water bottles using MWI at 10 GHz (Ricci et al. 2022).

MWI (mainly MWT) is a linear or non‐linear inverse method that requires a second detection step and is less accurate but faster than MWS. MWS can be more accurate and real‐time if MWS is used with an ML algorithm. For instance, contaminant detection in packaged foods was performed using MWS and ML by Urbinati et al. (2020). Their system employed an array of six 10 GHz antennas to identify metal spheres, glass, plastic spheres, triangular plastic, and cap shape plastic in safflower oil and hazelnut cocoa cream packaged in glass jars. The antennas were connected to a switching *S*‐matrix for each measurement in jars with and without contaminants, creating a labeled dataset in real time (3 ms) using MWS and ML for training. Deng et al. (2024) analyzed heavy metal (Pb) residues in different concentrations (50–0.03 mg/kg) in edible soybean oil that were detected by MWS at 2–12 GHz. The model demonstrated strong predictive performance with a root mean square error (RMSE) of 3.1654 mg/kg, a coefficient of determination (*R* ^2^) of 0.9605, and a relative prediction deviation (RPD) of 5.0479.

Overall, MWI and MWS are viable, low‐cost, and low‐power solutions for detecting a wide variety of physical contaminants (glass, plastic, metal, stones, copper wires, and dielectric rods) and even chemical residues (e.g., heavy metals) in both solid and liquid food products (contained in plastic or glass or uncontained). These techniques are effective across diverse food matrices and support non‐destructive, in‐line inspection, making them highly suitable for real‐time industrial applications to ensure food safety and quality.

#### 3.1.2 Microorganisms and Their Toxins

Pathogenic microorganisms such as *Escherichia coli, Salmonella*, and *Listeria* in food represent a serious risk to human health. Moreover, a high amount of microorganisms (total mold and yeast, total aerobic or anaerobic bacteria) can spoil and deteriorate food. Oberoi et al. (2012) detected 1–2 colonies forming uniform *E. coli* in water using MWS (cavity) at 7–8 GHz in 4–5 min, measuring relative permittivity. The total bacterial count of raw goat's milk was evaluated with an OECP as an MWS system by Zhu et al. (2018). For this purpose, the dielectric constant (*ε′*) and dielectric loss factor (*ε*″) were obtained from 77 hygiene‐qualified and 73 hygiene‐disqualified milk samples. As a result, the number of bacteria directly correlates with the solution's dielectric constant (Zhu et al. 2018). Hygiene‐disqualified milk with more bacteria had higher ε′ and ε″ at low frequencies. Probably, bacteria decompose sugars into conductive species, improving medium conductivity, and ionic conduction dominates dielectric loss at the radio‐frequency range, and protein and lactose content increases due to dominant dipole polarization over the MW frequency range (Zhu et al. 2014, 2015). Preprocessing (SG, SNV, and SG + SNV), PCA, and SPA (neural network) models were established and used to reduce the dimension of spectra, and SVM‐PCA was the best model with a 100% accuracy rate (Zhu et al. 2018).

Microorganisms generate toxins due to stress conditions in food products (Kebede et al. 2012). Aflatoxins (B1, B2) are the most known toxins, which are produced by *Aspergillus* spp. MW detection techniques were used for rapid and low‐cost evaluation of toxins in food products. For instance, Xu et al. (2022) developed MWS techniques for aflatoxin B1 detection in wheat samples presenting different mildew levels. Free‐space methods were used with an antenna at 2.5–11.5 GHz frequency band, recording the transmission index. After data preprocessing (least square filtering, BOSS approach), PLS, SVM, ELM, and RF models were evaluated for the prediction of quantitative measurement of aflatoxin B1 concentration in wheat. The BOSS‐SVM model achieved the best prediction for MWS‐based grain quality monitoring, with an RMSE of prediction (RMSEP) of 2.8 µg/kg, *R*_P_^2^ of 0.97, and RPD of 5.7, demonstrating its effectiveness for rapid, on‐site detection of stored grain safety. Deng et al. (2023) integrated MWS at 3.5–12.5 GHz with a deep learning‐based multi‐task convolutional neural network (CNN) model for qualitative analysis of mildew and aflatoxin B1 contamination in wheat. The CNN model achieved 100% accuracy in mildew classification, demonstrating its robustness in fungal detection. Additionally, the fusion CNN model effectively predicted aflatoxin B1 levels with an RMSEP of 2.0138 µg/kg and *R*_P_^2^ of 0.9807, highlighting its potential for precise, non‐destructive mycotoxin assessment in food safety applications. Overall, the studies demonstrate the potential of MWS devices and deep learning in monitoring mycotoxin contamination in cereals (Xu et al. 2022; Deng et al. 2023) (Table 5).

Therefore, MWS demonstrates strong potential for rapid, multi‐analyte detection of microorganisms and their toxins in liquid and solid food, respectively. The fusion with AI models, especially deep learning, is a key enabler of high accuracy. However, further research is needed to improve detection in complex matrices, reduce dependence on computational preprocessing, and validate results across diverse operational environments. Standardization of protocols and interdisciplinary collaborations will be essential for industrial scaling. Moreover, although studies have focused on MWS approaches, future research should also explore the potential of MWI systems for microbial and chemical hazard detection, evaluating their capabilities in terms of spatial localization, penetration depth, and integration with AI‐driven classification frameworks.

### 3.2 MC Measurement

MC in food materials and agricultural products can be measured using various MWS techniques, including free‐space methods with horn antennas, microstrip patches, and waveguide methods, primarily leveraging ionic conduction and dipole relaxation as loss mechanisms at MW frequencies (Ryynänen 1995; Skierucha et al. 2012). The dipole polarization of water molecules is particularly significant at MW frequencies, and as the frequency increases, the water molecules struggle to realign with the electric field, leading to an increase in energy losses and dielectric loss factors (*ε*″) (Skierucha et al. 2012). This phenomenon is most pronounced beyond 2 GHz, which makes the frequency range of 2–10 GHz particularly suitable for MC detection in food and agricultural materials, as shown in Table 6.

Several studies have applied MWS to measure the MC in various materials such as wheat (Trabelsi et al. 1998), forage (Shrestha et al. 2017), wheat, rough rice, barley (Zhang et al. 2019), animal feed and de‐oiled cake (Bekal et al. 2020), grain (Javanbakht et al. 2021b), soybeans, rice, and peanut (Jiarasuwan et al. 2021). Most studies utilize the MWS free‐space method, which employs microstrip antennas or horn antennas, especially in the frequency range of 2–10 GHz. For example, the MC of Korean short‐grain rough rice was estimated using the MWS free‐space method and ANN with a prediction accuracy (*R* ^2^ = 0.986) and a standard error of performance (SEP) of 0.52% (Kim et al. 2002). In addition, dielectric properties (*ε*′ and *ε*″) of potatoes at various MCs were measured using the OECP method at 2.45 GHz (Chee et al. 2014), which showed an increase in dielectric properties with higher MC, although the measurements became more consistent at higher MC. Other studies, such as Trabelsi and Nelson (2016), compared MW moisture meters with standard oven‐drying methods for various grains, demonstrating that MW moisture meters reduced grading time by 60% and improved consistency and quality in peanuts. Moreover, the MW moisture meter showed an error of 0.53%, compared to 0.87% for the standard moisture meter. Additionally, Ma et al. (2023) used a handheld nanoVNA with two Vivaldi broadband antennas and chemometric methods (XGBoost, CARS, CARS–CV, PLS–CARS) for predicting the MC of peanuts at 2–3 GHz, achieving an *R* ^2^ value of 0.9990, an actual MC of 35.7%, and a maximum prediction error of 1.52%. Low‐cost MWSs, such as nanoVNAs, represent a promising and accessible solution for field applications in food analysis. However, their reduced sensitivity and stability relative to laboratory‐grade systems limit their effectiveness in high‐precision tasks. Further research involving calibration and direct performance comparisons with lab‐scale instruments is necessary to establish their reliability and suitability for food quality assessment.

Yigit and Duysak (2022) employed the free‐space method (1–2.48 GHz) with chemometric methods like KNN, SVR, and ANN for predicting MC (8%–25%) in flowing grains such as bulgur wheat, durum wheat, and corn silage kernels, with KNN offering the best predictions (*R* ^2^ = 0.99 and 0.98) for durum wheat and corn silage kernels and SVR offering the best model prediction (*R* ^2 ^= 0.89) for bulgur wheat. Further research by Yigit et al. (2024) developed a novel deep‐learning CNN architecture that outperformed pre‐trained models for detecting MC in flowing grains. Their CNN model achieved lower mean absolute error (MAE = 0.0411), mean squared error (MSE = 0.0149), and RMSE (0.122) compared to earlier architectures. The results indicate that CNN combined with signal processing can effectively determine the MC in grain samples in less time, with the new model completing its tasks in 325 s, a significant improvement over the previous 72‐min architecture. In addition, a study by Bai et al. (2024) successfully detected the MC of wheat using MWS with a microstrip antenna in the 2.5–11.5 GHz range, combining chemometric models like CARS, GA, and SVR. The CARS–GA–SVR combined model achieved a prediction accuracy with *R* ^2^ = 0.9756 and RPD = 6.3234.

*S*‐parameters and dielectric properties are key techniques for MWI and MWS to measure MC across 0.3–40 GHz. *S*‐parameters are preferred due to their contactless nature, simplicity, broad frequency range, and compact sensor size, making them highly effective for real‐time applications (Javanbakht et al. 2021b). Although dielectric constant measurements remain accurate up to the V‐band (below 100 GHz), input impedance variations are more suitable for low‐frequency bands (below S‐band) in grain and mineral material analysis.

Overall, the reviewed studies demonstrate that both MWI and MWS techniques can be effectively applied to determine the MC of solid food products. In addition to research prototypes, commercially available microwave‐based instruments such as the Sartorius LMA200, TEWS Elektronik sensors, MicroRadar, and MAC Instruments analyzers offer rapid, non‐destructive, and real‐time MC assessment. These technologies significantly improve process efficiency and quality control in a wide range of applications across the food (solid, liquid, fluid, and bulk) and agricultural sectors, as summarized in Table 3.

### 3.3 Adulteration Detection

Food fraud and adulteration are aimed for misleading consumers, intentional fraud, and unjust financial gain. Thus, food authentication is crucial for food safety, quality, and consumer protection, as well as national legislation and international standards. Researchers have concentrated on developing non‐destructive, practical (e.g., hand‐held), and real‐time techniques to prevent food fraud. Some examples of honey, milk, and other food adulterations are shown in Table 7.

#### 3.3.1 Honey

Honey is a food product that can be the most exposed food item for adulteration due to its high price. OECP has been mainly used to detect honey adulteration in MW technology. Pure jujube honey, yellow‐locust honey, and milk‐vetch honey were analyzed by OECP at 10–4500 MHz for detection of adulteration with water (Guo et al. 2010) and sucrose syrup (Guo et al. 2011). Guo et al. (2010) revealed that the dielectric constants of pure honey and honey with water‐added samples decrease with frequency and water content. They found strong correlations between dielectric constant and soluble solids and water contents. Guo et al. (2011) also found a significant negative correlation between the dielectric loss factor and sucrose content in all honey–sucrose syrup mixtures and the dielectric relaxation in pure honey, pure sucrose syrup, and honey–sucrose syrup mixtures. The syrup is a mixed liquid with varying electrolyte content, capable of ionizing positive and negative ions in water, demonstrating conductivity (Liu et al. 2022). Similarly, at 1–20 GHz frequencies, water (0%–80%) adulterated honey was successfully detected by OECP (Yakubu et al. 2019). As water content increased, the loss factor of the honey–water mixture decreased, resulting in a decrease in the mineral nutrients or vitamins of pure honey. The studies suggest that MW dielectric properties can be used to detect sucrose‐ and water‐adulterated honey or sense sucrose content in honey, suggesting potential marketplace applications.

#### 3.3.2 Milk

Several studies have demonstrated adulteration detection in milk by measurement of dielectric properties at certain frequencies in a range of 10 MHz–20 GHz, as shown in Table 7. Water and urea were detected in raw milk adulteration. For instance, Guo et al. (2010) investigated the detection of water adulteration and freshness in raw cow's milk during storage at 22°C, 36 h and 5°C, 144 h by using an OECP technology at radio and MW frequencies at a range of 10–4500 MHz. Results showed that raw milk had the lowest dielectric constant and highest loss factor at frequencies above 20 MHz and the highest linear coefficient of determination (*R* ^2^ = 0.995) at 915 MHz between milk concentration and loss factor. Similarly, Jitendra Murthy et al. (2017) studied the detection of freshness and adulteration (with water) in raw cow's milk at MW frequencies at 10–4500 MHz using an X‐band bench and OECP probe. The milk's dielectric constant decreased with frequency increase, reaching a minimum at 1700 MHz in the 10–4500 MHz frequency range at 22°C. Raw milk exhibited the lowest dielectric constant at frequencies >20 MHz and the highest loss factor compared to diluted milk. Eventually, the penetration depth in milk samples increased with decreasing frequency, water content, and storage time (up to 144 h), enabling the detection of dielectric properties changes. Another study found that polar molecule (water and urea) adulteration in whole milk and skim milk was detected by MW dielectric spectroscopy with an OECP at specific 17 GHz from the range between 100 MHz and 20 GHz (Zhao et al. 2019).

#### 3.3.3 Other Food Adulterations

Sunflower and canola oil adulteration in sesame oil was detected by using dielectric properties with the radiofrequency range at 40 kHz–20 MHz, combined with chemometric (ANN) (Soltani Firouz et al. 2022). As a result, the ANN classifier showed 100% perfect accuracy and quantification of sunflower oil, canola oil, and sunflower + canola oils, with *R* ^2^_Test_ of 1, 1, and 0.9999, respectively. Thus, although not in the MW range, classification and quantification of oil adulteration can be detected using dielectric properties. Besides, at 2.5–11.5 GHz, wheat flour adulterated with talcum powder was detected with the MWS free space method by using two antennas together with classification and regression models (Xu et al. 2024). The results show that the SSA‐XGBoost classification model had high prediction accuracy (100%) and *R* ^2^ = 0.98.

In conclusion, food adulteration has been effectively detected using MWS techniques—primarily the OECP and free‐space methods. The existing studies confirm the success of MWS in identifying adulterants in both solid and liquid food samples. However, further research is needed to expand the scope of these studies and enhance their reliability through integration with ML, which can help develop robust predictive models for broader applications. Notably, to the best of our knowledge, there is currently no published research on the use of MWI systems for food adulteration detection. Therefore, future studies should be encouraged to explore the potential of MWI, particularly for industrial‐scale implementation and real‐time inspection purposes.

### 3.4 Food Quality Control

Food safety and quality are crucial for consumers, requiring strict legislation and compulsory examination. Non‐destructive, active, and quick testing techniques are essential for controlling food quality and safety. Developing non‐destructive techniques allows for measurement and analysis of food parameters, reducing waste and allowing repeated measures over time. MWI and MWS technologies have been used for this purpose, as shown in Table 8. For instance, the deterioration of milk (whole, low fat, and skim milk) at 17–20°C in 2 weeks (Nunes et al. 2006), the freshness of milk (Guo et al. 2010), quality defects (Castro‐Giráldez et al. 2010), and water‐holding capacity (Abdullah et al. 2014) of meat were evaluated using MW technology for food quality control. Iaccheri et al. (2022) detected the concentration of sodium chloride, citric acids, saccharose of egg products; Brix of fruit juices; temperature; and concentration of milk with MWS (OECP) and chemometric (PLSR). The results were in *R* ^2^ = 0.933–0.988. Besides, Tai et al. (2020) reported that MWI (waveguide antenna) at 7–13 GHz detected thin albumen, thick albumen, and yolk of eggs for evaluating egg health. Kizilay et al. (2023) detected glued‐unshelled, healthy‐unshelled, and rancid walnuts using MWI at 7–12 GHz and ANN modeling using *S*_11_ and *S*_22_ parameters, resulting in minimum errors in range of 7.33%–16.92%.

MW technology is also found useful for assessing fruit quality, determining ripeness and distinguishing characteristics, and determining the internal quality of fruits (Garvin et al. 2023). The maturity of the watermelon was determined by MWI using a circular array with ten Vivaldi antennas (Garvin et al. 2023). The images detect internal characteristics of watermelon, including water and sugar concentration and distribution, with high permittivity categorized by red heat signatures. Thus, maturity was evaluated quickly, and accurate maturity was determined with validation by the sugar concentration measurements. Similarly, Choffin et al. (2025) reported watermelon ripeness (86% accuracy) with MWI and CNN algorithms (threefold cross validation).

Freshness (fresh or stale) of potato and apple was identified with waveguides at 4.9–7.05 GHz, using permittivity (*ε*′) (Ates et al. 2017). Seeds in lemons and grapefruits were detected by MWI using a radar algorithm (Ghavami et al. 2019). Huygens principle‐based method used the difference in the dielectric properties between the fruit and its seeds (dielectric variation) to capture the contrast, detect internal seeds, and distinguish between seeded and seedless products. For sorting and grading, fruits (apple, avocado, dragon fruit, guava, and mango) were evaluated by transmitting and receiving antennas (at 932 MHz) with the measurement of *S*‐parameters (*S*_11_ and *S*_21_) and combined with ML algorithms (KNN) and neural network (Tran et al. 2023). Results showed that neural network and KNN models had high accuracy (98.75% and 99.75%, respectively) for fruit classification. For ripening classification, KNN was more effective (98.4%) than neural network (96.6%) (Tran et al. 2023). Egg freshness was detected with a waveguide (0.9–1.7 GHz) combined with chemometric methods PLS and ANN regression and SIMCA and ANN classification for determining storage duration (Akbarzadeh et al. 2019). Results showed that the best predictive models were obtained from ANN analysis, and the total accuracy of 100% was obtained from SIMCA and ANN classification of eggs using *S*_11_ input spectra. Thus, ANN was able to predict the quality indices of shell eggs, and SIMCA classified the eggs based on storage time with MWS (waveguide) detection (Akbarzadeh et al. 2019).

Soluble solids content (SSC), TA, and consistency (Bostwick) in both dilution and evaporation conditions (with different temperatures and pressure) for tomato paste were observed with PPs in‐line sensor combined with PLS and iPLS methods (Zhang et al. 2014). The chemical properties of food products affect their dielectric properties, which enables MWS to measure the properties of tomato pastes. As a result, the estimation of SSC, TA, and Bostwick consistency of tomato paste was accurate with iPLS and PLS (*R* > 0.92). Tang et al. (2024) demonstrated that SSC and hardness of pears were detected OECP using *ε*′ and *ε*″ parameters, combined with PLSR, SVR, and particle swarm optimization–least squares support vector regression (PSO–LSSVR), and PLSR was the best prediction method among all tested. For *ε*′ and hardness, *R* ^2^ and MSE were 0.77 and 0.073, respectively, and for *ε*″ and SSC, *R* ^2^ and MSE were 0.91 and 0.087, respectively (Tang et al. 2024).

In conclusion, MWS and MWI are effective, non‐destructive techniques for assessing food quality attributes such as freshness, composition, ripeness, and internal defects. MWS, often used with chemometric models, is successful in analyzing liquids and solids, whereas MWI offers spatial insights for classification and sorting tasks. Integrating these methods with ML significantly improves accuracy. Overall, MWS and MWI show strong potential for real‐time food quality monitoring, though further research is needed to optimize industrial application and expand the use of MWI in areas like adulteration detection.

#### 3.4.1 Food Composition Detection

Food quality control is greatly improved by the application of food composition detection technologies, which enable precise determination of proximate composition, including moisture, protein, fat, and ash content. Numerous studies have explored the efficiency of MW technologies in this context, as summarized in Table 9. These studies may demonstrate the potential of MW‐based methods to provide rapid, non‐destructive, and accurate analyses, making them valuable tools for ensuring food quality and safety. For instance, a study investigated the dielectric constant of a 10:1 mix solution of proteins and papain enzyme using an MW surface imaging technique. Results show a decrease in dielectric constant when proteins are added, providing insight into protein interaction (Urvashi et al. 2019). MWS resonator detected water activity, salt content, and water content in dry‐cured ham using amplitudes *S*_11_ and *S*_21_, indicating its potential for determining ham quality (*R* ^2^ = 0.76–0.98) (Bjarnadottir et al. 2015). Moreover, MW waveguide at 1.6–2.7 GHz successfully detected fructose, ethanol, sodium chloride in water, fat in milk, and carbohydrate and water content in fruit juices, with *R* ^2^ of 0.999, 0.991, and 0.990, respectively (Ragni et al. 2017). The low‐range MW spectra instrument, with inexpensive electronic components, could be a valid solution for determining food compositional features in quality control processes, potentially allowing online use. Besides, the fat and protein content of whole milk, semi‐skimmed milk, and skimmed milk were evaluated with resonator cavities using *S*_11_ and *S*_21_ parameters (Joshi et al. 2017). As a result, they evaluated that this method classifies milk types based on fat and protein content (*R* ^2^ = 0.98 and 0.949, respectively), providing a platform for composition checks and preventing spoilage or adulteration. Urvashi et al. (2019) evaluated papain enzyme activity in a mixed solution of protein (egg white, egg yolk, plant protein, and insulin) with fork‐type OECP at 5.5–7.5 GHz, measuring *ε*′. As a result, *ε*′ was decreased by the addition of papain enzyme to protein. Thus, the authors explored the potential of understanding protein–enzyme interactions through a focus on dielectric properties. Moreover, Basaran et al. (2010) studied the dielectric properties (*ɛ*′ and ɛ″) of chicken and fish muscle treated with microbial transglutaminase. They found that transglutaminase in restructured chicken breast, salmon, and trout muscle products had dielectric properties over 20–130°C by using OECP at 433, 915, and 1800 MHz.

Zhao et al. (2016) also studied the fat content of ground beef by using MWS and NIR spectroscopy combined with chemometric (MSC, SNV, PCA, and PLSR). For MWS, the OECP technique was used at 0.2–20 GHz, measuring of *ɛ*′, *ɛ*″, and loss tangent. Prediction models for fat content in ground beef samples were the best performance for MWS, using dielectric spectral data (*ɛ*′), with *R* ^2^ = 0.87 and RMSEP = 2.71% w/w. Thus, the time‐domain reflectometry MWS can effectively control the quality of ground beef products. However, the best predictive model for NIR spectroscopy on ground beef samples achieved an *R*_P_^2^ value of 0.99 and RMSEP of 0.71% w/w after applying the Martens uncertainty test (Zhao et al. 2016). On the other hand, the lactose content of whole and skim milk and the solids‐not‐fat content of skim milk were identified with the OECP technique (Liu et al. 2018). *ε*′ had a weak positive linear relationship with lactose content for whole milk but had a negative linear relationship with skim milk. *ε*′′ had a strong negative linear relationship with lactose content at <1 GHz and had a strong positive linear relationship at >2.3 GHz. Moreover, ε′ and solids‐not‐fat content *R* ^2^ > 0.940 below 50 MHz and *R* ^2^ = −0.990 above 150 MHz. *ε*″ and solids‐not‐fat content *R* ^2^ > 0.970 at all investigated frequencies. Besides, the penetration depth of EM waves in milk decreased with increased frequency or solids‐not‐fat content (Liu et al. 2018). Moreover, different concentrations of 50–0.03 mg/kg of Pb in edible soybean oil were observed using MWS at 2–12 GHz and CNN and residual attention CNN algorithms (Deng et al. 2024). They found that the performance of the model was a great prediction (3.1654 mg/kg RMSE, 0.9605 *R* ^2^, and 5.0479 RPD). Thus, heavy metal levels were detected in edible oils with MWS and chemometrics.

MWS and MWI techniques are effective, non‐destructive tools for detecting food composition, including moisture, fat, protein, ash, salt, water activity, sugar, ethanol, and even heavy metal content across various food matrices. MWS, particularly with OECP, resonators, and waveguide systems, has demonstrated strong predictive capabilities, with high correlation coefficients (*R* ^2^ > 0.90) for most compositional analyses. Applications span diverse products such as milk, fruit juices, dry‐cured ham, ground beef, and edible oils, with some studies integrating chemometric and ML models (PLSR, CNN, etc.) to enhance accuracy and reliability. These models effectively captured dielectric changes corresponding to chemical variations, such as enzyme activity, fat content, and lactose concentration, even under varying frequencies and temperatures. Overall, MWS/MWI technologies show strong promise for real‐time quality control and online food monitoring, offering low‐cost, scalable solutions. Further development and validation, particularly in industrial environments, will strengthen their role in modern food composition analysis and safety assurance.

## 4 Advantages and Limitations of MWS and Imaging Systems for Food Applications

MWS and MWI technologies have emerged as highly promising tools for assessing food quality and safety. This section highlights the advantages and limitations of MWS and MWI systems for food applications. Furthermore, these technologies are compared with conventional methods such as metal detectors (MD), x‐ray imaging (XRI), and terahertz (THz) imaging, offering a comprehensive overview of their respective strengths and challenges.

### 4.1 Advantages of MWS and Imaging Systems

One of the key advantages of MWI and MWS (free‐space method) is its **non‐destructive and safe nature to users (**Choffin et al. 2025). Unlike XRI, which emits ionizing radiation and may pose health risks to operators, MWI utilizes non‐ionizing, low‐intensity radiation, making it a safer alternative for food inspection (Tang et al. 2022). Additionally, its non‐contact nature ensures that food products remain unaltered during analysis, allowing real‐time monitoring without compromising quality (Gartshore et al. 2021).

In terms of **detection capabilities**, MWI excels in identifying contaminants that are challenging for traditional imaging methods. For instance, it can effectively detect plastic, glass, bone, and cartilage fragments in meat and poultry products, which are often invisible to XRI and THz imaging systems (Urbinati et al. 2020; Ricci et al. 2021; Tang et al. 2022; Vp and Susan 2023). Furthermore, MW signals in the GHz frequency range penetrate up to a few centimeters, enabling the inspection of non‐metallic food packaging, a limitation observed in MD and XRI technologies (Tang et al. 2022).

Another advantage of MW systems is their **cost‐effectiveness and adaptability to industrial settings**. Compared to MRI and other high‐end imaging technologies, MW‐based equipment is relatively inexpensive, with some devices, such as resonator cavity sensors, costing as little as $75 to manufacture (Li et al. 2022). The simple mechanical structure of MW systems facilitates their integration into food production lines, where they can operate in offline, at‐line, online, and inline modes, allowing real‐time analysis at various processing stages (Bellizzi et al. 2022; Zeni et al. 2023).

MW systems also offer **high sensitivity to moisture and food composition analysis**. MW waves interact strongly with water‐containing materials due to their high permittivity, making MW‐based methods particularly useful for assessing moisture levels and determining food composition (Tang et al. 2022). This property is valuable in applications in liquid, semi‐solid, and solid food products (i.e., high‐moisture as well as low‐moisture) where MW technology provides stable and accurate results (Liu et al. 2022).

### 4.2 Limitations of MWS and Imaging Systems

Despite its numerous advantages, MWI faces several technical and practical limitations that must be addressed for broader industrial adoption. One of the primary challenges is the **complexity of analyzing heterogeneous food samples**. Even a single food item exhibits distinct electrical properties due to non‐homogeneous structure, such as variations in MC (Zeni et al. 2023). Additionally, the presence of multiple compositional constituents with varying dielectric properties creates a complex scattering environment, affecting detection accuracy.

Moreover, most existing studies focus on solid or semi‐solid foods, with minimal exploration of dynamic or heterogeneous systems. Although the MW systems have demonstrated the potential to detect certain low‐abundance contaminants like aflatoxins (down to 2.8 µg/kg), there is insufficient evidence regarding their sensitivity for detecting trace levels of pesticides. This highlights a significant research gap and an opportunity for future studies to expand the applicability of MW systems to more diverse food systems and contaminants.

Another critical limitation is the **impact of temperature and frequency selection**. Temperature fluctuations can influence the dielectric properties of food, leading to measurement inconsistencies (Chandra et al. 2015). The mechanism by which temperature fluctuations affect dielectric properties is primarily attributed to changes in the mobility of polar molecules, particularly water, within the food matrix. As temperature increases, molecular relaxation times decrease, resulting in variations in both the dielectric constant and dielectric loss factor (Nelson and Bartley 2000). These changes can significantly impact the accuracy of MW‐based measurements. To mitigate this issue, mathematical models such as the Debye or Cole‐Cole model have been employed to characterize the frequency‐dependent behavior of food materials (Chandra et al. 2015). In practical applications, temperature sensors and reference materials should be incorporated into the system to provide real‐time correction during data acquisition.

MWI encounters challenges related to **acquisition speed and algorithm complexity**. Compared to conventional imaging systems, MW‐based methods generally exhibit lower data acquisition speeds, which may limit their application in high‐throughput production environments (Tang et al. 2022). Additionally, the EM inverse scattering problem, which forms the basis of MWI, involves solving highly non‐linear equations, making the image reconstruction process computationally intensive (Tang et al. 2022).

Moreover, in medical applications, MWS and MWI have a **low spatial resolution** compared to XRI and MRI (Chandra et al. 2015; Kiran 2020). Besides, recent advancements in imaging algorithms, data acquisition systems, and enabling technologies have further enhanced its performance, particularly in applications such as breast cancer detection (Halim et al. 2022).

From a system and experimental standpoint, MWI requires high dynamic range measurement setups to **detect weak scattering signals**. Efficient coupling of MW power into food samples is also critical to optimize penetration depth and spatial resolution. Moreover, large‐size food products require advanced three‐dimensional (3D) imaging models that account for the effects of antennas and feeding networks, further increasing system complexity (Chandra et al. 2015). Moreover, in high‐moisture foods, MWI faces signal attenuation due to water's high dielectric loss, which limits penetration and weakens defect signals. This challenge can be mitigated through advanced signal processing, inversion algorithms, and optimization of operating frequencies, including wideband or multi‐frequency techniques.

## 5 Conclusion

MW sensing and imaging systems offer a promising solution for food quality assurance, providing safe, non‐destructive, cost‐effective, and highly sensitive detection capabilities. These technologies may overcome limitations of traditional inspection methods, particularly in detecting non‐metallic contaminants. However, challenges such as sample heterogeneity, temperature sensitivity, algorithm complexity, and system‐level limitations must be addressed to enhance practical implementation. With continuous advancements in computational imaging models, MWI has the potential to become integrated with standard techniques for real‐time food inspection in high‐speed industrial production lines. Future research should focus on improving signal processing with AI, developing low‐cost portable systems, and conducting large‐scale validation in real‐world food applications. This will help bridge the gap between lab innovation and industrial use.

## Author Contributions

**Aysenur Betul Bilgin**: investigation, conceptualization, writing – original draft, visualization. **Pervin Basaran**: writing – review and editing, supervision.

## Conflicts of Interest

The authors declare no conflicts of interest.
